# Endogenous osteoprotegerin (OPG) represses ERα and promotes stemness and chemoresistance in breast cancer cells

**DOI:** 10.1038/s41420-024-02151-8

**Published:** 2024-08-24

**Authors:** Noura N. Alraouji, Dilek Colak, Falah H. Al-mohanna, Ayodele A. Alaiya, Abdelilah Aboussekhra

**Affiliations:** 1 Department of Molecular Oncology, King Faisal Specialist Hospital and Research Center, Riyadh, 11211 Saudi Arabia; 2 Department of Comparative Medicine, King Faisal Specialist Hospital and Research Center, Riyadh, 11211 Saudi Arabia; 3 Department of Cell Therapy & Immunobiology, King Faisal Specialist Hospital and Research Center, Riyadh, 11211 Saudi Arabia

**Keywords:** Diseases, Cancer

## Abstract

Breast cancer (BC) is the most prevalent cancer and the leading cause of death among women worldwide. The osteoprotegerin (OPG) cytokine, a decoy receptor for RANKL and a key player in bone homeostasis, has pro-and anti-carcinogenic effects in various types of cancer, including breast neoplasms. In the present study, we have shown that ectopic expression of OPG in breast epithelial/cancer cells promotes the pro-metastatic processes epithelial-to-mesenchymal transition (EMT), stemness, angiogenesis as well as the activation of breast stromal fibroblasts. Furthermore, proteomics analysis, which allows the identification and quantification of a plethora of known and unknown proteins, has shown a strong and significant correlation between OPG upregulation and the expression of proteins with functions in EMT and stemness. On the other hand, OPG knockdown in triple-negative breast cancer (TNBC) cells inhibited the formation of cancer stem cells. Importantly, while OPG upregulation significantly enhanced the resistance of luminal BC cells to cisplatin and docetaxel, OPG downregulation sensitized TNBC cells to these chemotherapeutic drugs. We have also shown that OPG negatively controls estrogen receptor α (ERα), and OPG upregulation correlated well with the expression of genes related to ER-negative claudin low cells. Collectively, these results show that OPG promotes stemness and the consequent chemoresistance of breast cancer cells.

## Background

Breast cancer (BC) is the most prevalent form of cancer diagnosed among women worldwide [1]. Despite the development of several treatment modalities and precision therapeutics, tumor resistance, recurrence, and metastasis remain very frequent among high proportion of these patients [2]. Several lines of evidence indicate that the main reason for these anti-cure obstacles is the presence of a small population of cells with stemness features called breast cancer stem cells (BCSCs). These cells have several characteristics, including self-renewal ability, the capability to differentiate into different lineages, the capacity to evade cell death, and also the ability to metastasize [3, 4]. The presence of a high proportion of BCSCs is associated with disease progression, tumor aggressiveness, and drug resistance in breast cancer [4, 5]. Therefore, there is an urgent demand to further characterize these cells and identify the main factors responsible for promoting stemness. Osteoprotegerin (OPG) is one of these factors, which could potentiate stemness features in breast cancer cells. OPG is a secreted glycoprotein belonging to the tumor necrosis factor receptor (TNFR) superfamily. OPG was first identified and named for its function in bone remodeling and homeostasis [6]. In this context, OPG is acting as a decoy receptor for another TNF superfamily member Receptor Activator of NF-κB Ligand (RANKL; TNFSF11), and blocks its interaction with RANK (TNFRSF11A), and thereby prevents the stimulation of osteoclast maturation and bone resorption [6, 7]. In contrast, subsequent investigations indicated that OPG also acts as a decoy receptor by binding to another TNF superfamily member, TNF-Related Apoptosis Inducing Ligand (TRAIL; TNFSF10), and thus prevents TRAIL-induced apoptosis in cell-expressing OPG [8]. Thereby, OPG may also have a protective anti-apoptotic effect, and acts as an endocrine survival factor in cancer cells by overcoming the tumor surveillance exerted by TRAIL [9]. Consistently, OPG expression level was found higher in breast cancer cells and tissues [10–12]. Furthermore, a higher level of serum OPG was associated with higher mortality rates and poorer overall survival, especially in patients with ERα−positive disease [13, 14]. In addition, OPG stimulates breast cancer cell metastasis in vivo [15]. Thereby, the exact role of OPG in breast cancer remains controversial, but it seems more likely that endogenous OPG promotes breast carcinogenesis. Therefore, in the present report, we sought to investigate the role of this cytokine in breast carcinogenesis, and the acquisition of stemness features. We have shown OPG-dependent promotion of the pro-metastatic processes epithelial-to-mesenchymal transition (EMT) and stemness in breast epithelial and cancer cells.

## Results

### OPG upregulation promotes breast carcinogenesis and downregulates ERα

**Fig. 1:**
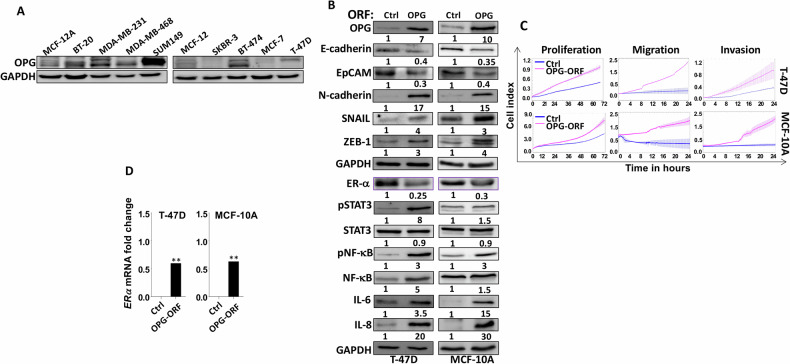
OPG upregulation initiates the EMT process in breast epithelial cells. **A** Western blot images with GAPDH used as internal control. **B** T-47D and MCF-10A cells were transfected with either an empty vector (Ctrl) or a plasmid bearing the OPG‐ORF (ORF). Whole cell lysates were used for immunoblotting analysis. The numbers underneath each band represent fold changes relative to the control (Ctrl) after normalization to GAPDH. Phosphorylated protein fractions were determined by quantification and normalization against their relative non-phosphorylated forms. **C** xCELLigence RTCA System was used for assessing the abilities of the indicated cells in a real-time manner. **D** Real-time RT‐PCR to assess the expression level of the *ERα* gene. Error bars represent mean ± SEM (*n* = 3). **P ≤ 0.01.

We have first assessed the level of the OPG protein in eight different breast cancer cell lines and two breast non-carcinogenic epithelial cell lines. To this end, whole cell lysates were prepared and used for immunoblotting analysis using specific antibodies. Figure 1A shows that OPG is highly expressed in the triple-negative breast cancer (TNBC) cell lines (BT-20, MDA-MB-231, MDA-MB-468, and SUM149PT), relative to the other breast cancer cell lines (SKBR-3, MCF-7 and T-47D) and the non-carcinogenic breast epithelial cells (MCF-12A and MCF-10A). This suggests a potential role of OPG in the aggressive features and mesenchymal characteristics of TNBC cells. Therefore, we sought to investigate the role of OPG in breast carcinogenesis and its possible association with ERα expression. To this end, we ectopically expressed OPG in breast epithelial cells by introducing a plasmid bearing the OPG-open reading frame (ORF) into T-47D, MCF-10A, and MCF-7 cell lines (OPG-ORF), while the corresponding empty vector was used as a control (Ctrl). As expected, the level of the OPG protein was increased in OPG-expressing cells compared with control cells (Figs. 1B, and Fig. S1A). Concomitantly, the epithelial markers E-cadherin and EpCAM were downregulated and the mesenchymal markers N-cadherin, Snail and ZEB1 were markedly upregulated in OPG-expressing cells (Fig. 1B and S1B). This potential OPG-dependent EMT induction was confirmed by showing higher cell proliferation, migration and invasion abilities of OPG-expressing cells compared with controls (Fig. 1C, and S1C). Furthermore, the upregulation of OPG activated two important BC-related pathways, STAT3 and NF-κB as well as their downstream mediators IL-6 and IL-8 (Fig. 1B, and Fig. S1A). These results show that OPG upregulation in breast epithelial cells induces the EMT process and activates the pro-carcinogenic STAT3 and NF-κB pathways. Because OPG was lowly expressed in ΕRα-positive cells, we explored the effect of OPG upregulation on the expression of ΕRα. Interestingly, OPG upregulation reduced the expression of ERα in the breast cancer cells (MCF-7 and T-47D) and the non-tumorigenic breast epithelial cells (MCF-10A), which express low levels of ΕRα (Figs. 1B, and S1A). This effect was also observed at the mRNA level (Figs. 1D, and S1D). This indicates that OPG upregulation promotes the EMT process and reduces ΕRα expression.

### OPG upregulation promotes stemness features in breast epithelial cells

**Fig. 2:**
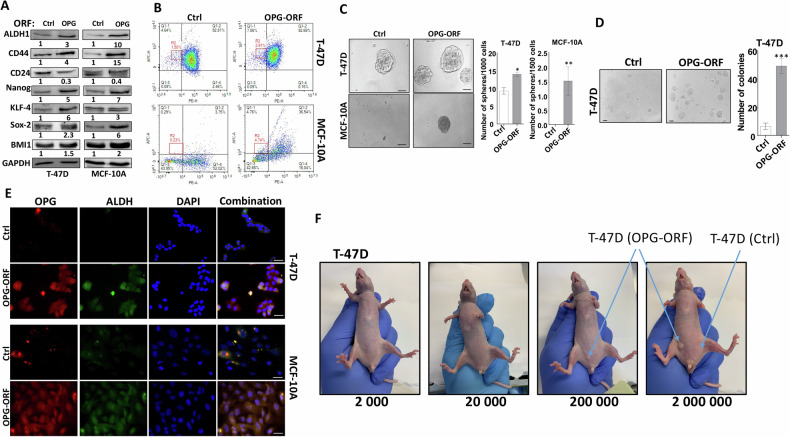
OPG upregulation promotes the stemness features of breast epithelial cells. **A** Western blot images with GAPDH used as internal control. The numbers underneath each band represent fold changes relative to the control (Ctrl) after normalization to GAPDH. **B** Cells (2 × 10^5^) were double‐stained for both CD24 and CD44, and the proportions of the subpopulation CD44^high^/CD24^low^ were determined by flow cytometry and are shown in the boxes. **C** Cells (1 × 10^3^) were seeded in ultra-low attachment 96‐well plate containing stem cell‐specific medium (100 μl). The number of spheroids (>100 μm) were counted. Representative photographs of spheroids (left panel), number of formed spheroids (right panel). Error bars represent mean ± SEM (*n* = 3). *P ≤ 0.05; **P ≤ 0.01. **D** Cells were plated in soft agar, and after 7 days, the number of colonies ( >100 μm) were counted. Representative photographs of colonies (left panel), number of formed colonies (right panel). Scale bars represent 100 μm. Error bars represent mean ± SEM (*n* = 3). ***P ≤ 0.001. **E** Immunofluorescence, photographs were taken by fluorescent microscope. Scale bars represent 25 μm. **F** Different concentrations (2 × 10^3^, 2 × 10^4^, 2 × 10^5^, 2 × 10^6^, *n* = 3 for each cell concentration) of T-47D (OPG-ORF and Ctrl) cells were injected under the right and left nipple of female nude mice, respectively.

Next, we decided to check whether OPG-mediated EMT induces stemness as well. Immunoblotting analysis revealed that the ectopic expression of OPG resulted in marked upregulation of the major BCSC markers ALDH1 and CD44, whereas CD24 was downregulated in T-47D, MCF-10A, and MCF-7 cells compared with their corresponding controls (Figs. 2A, and S1A). Concomitantly, OPG upregulation induced the expression of other important stemness markers, namely Sox2, Nanog, KLF4, and BMI1 (Fig. 2A). These results indicate that OPG promotes stemness features in breast epithelial cells. To confirm this, we assessed by flow cytometry, the proportion of CD44^high^/CD24^low^ subpopulation. Figure 2B shows an increase in the proportion of CD44^high^/CD24^low^ subpopulation in OPG-expressing T-47D and MCF-10A cells compared with their respective control cells. In addition, we examined the effect of OPG upregulation on the self‐renewal potential of breast epithelial cells using an in vitro spheroid formation assay. Figure 2C and Supplementary Fig. S1B show that the ectopic expression of OPG significantly increased the number of spheroids in T-47D, MCF-10A, and MCF-7 cells compared with their respective controls. Furthermore, while control cells formed few colonies in soft agar, OPG-expressing cells formed a significantly higher number of colonies (Fig. 2D). To further confirm the link between OPG expression and stemness characteristics in breast epithelial cells, a co-staining immunofluorescence experiment was performed. Figure 2E shows that all cells expressing high levels of OPG also stained positive for ALDH1. This confirms the OPG-dependent upregulation of ALDH1 in breast epithelial cells, and the OPG-dependent promotion of stemness.

To demonstrate this, the in vivo limiting dilution tumor initiation assay was performed by injecting T-47D cells expressing either OPG-ORF or a control vector at different concentrations (2 × 10^3^, 2 × 10^4^, 2 × 10^5^, 2 × 10^6^) under the right and left nipples of female nude mice, respectively. After 5 months, while control cells did not form tumors, OPG-expressing cells generated orthotopic tumor xenografts only when the number of injected cells reached 2 × 10^5^ and 2 × 10^6^, which generated tumors with sizes proportional to the number of the injected cells (Fig. 2F). furthermore, MCF-7 cells expressing either OPG-ORF or a control vector (5 × 10^6^) were injected under the right nipple of female nude mice (*n* = 5 for each inoculation). While OPG-expressing cells were able to generate orthotopic tumor xenografts in 2 out of 5 mice, control cells did not generate any tumors (Fig. S2). Together, these results show that OPG upregulation promotes stemness in breast epithelial cells, both cancerous and non-carcinogenic.

### OPG upregulation promotes stemness in breast epithelial cells through the Wnt/β-catenin pathway

**Fig. 3:**
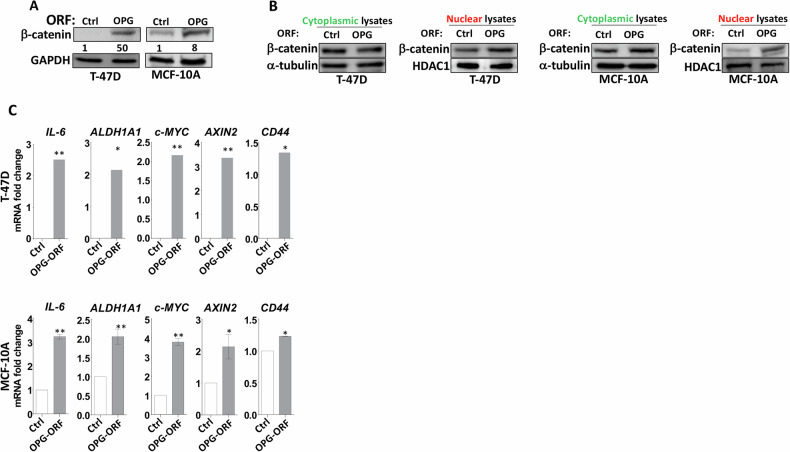
OPG induces the stemness features in breast epithelial cells through the Wnt/β-catenin pathway. **A** Western blot images with GAPDH used as internal control. **B** Cytoplasmic and nuclear lysates were prepared for immunoblotting analysis. The numbers underneath each band represent fold changes relative to the control (Ctrl) after correction against specific internal controls. **C** qRT‐PCR, Error bars represent mean ± SEM (*n* = 3). *P ≤ 0.05; **P ≤ 0.01.

To delineate the molecular pathway that mediates OPG-dependent promotion of stemness in BC cells, we first tested the effect of OPG upregulation on the important stemness-regulator pathway β-catenin. Figure 3A shows that OPG upregulation enhanced the expression of β-catenin both in T-47D and MCF-10A cells. Since β-catenin is a nuclear protein, we tested the effect of OPG upregulation on the β‐catenin translocation to the nucleus. To this end, the level of this transcription factor was assessed in the nuclear and cytoplasmic fractions prepared from OPG-expressing cells and their corresponding controls. The immunoblots showed that the ectopic expression of OPG increased the nuclear β‐catenin level in both T-47D and MCF-10A cells compared with their respective controls, whereas the cytoplasmic levels of β‐catenin were only slightly affected by OPG upregulation (Fig. 3B). Therefore, OPG upregulation enhances the accumulation of β‐catenin into the nucleus of breast epithelial cells. This result was confirmed by showing that the ectopic expression of OPG transactivates the β‐catenin target genes *ALDH1A1*, *CD44*, *AXIN2*, *c-MYC*, and *IL-6* (Figs. 3C and S1C). These results indicate that OPG is a potent inducer of the Wnt/β‐catenin signaling pathway, and therefore could enhance the stemness features through this CSC-regulatory pathway in addition to the STAT3/NF-κB pathway.

### OPG upregulation affects the expression of several genes involved in various physiological processes

**Fig. 4:**
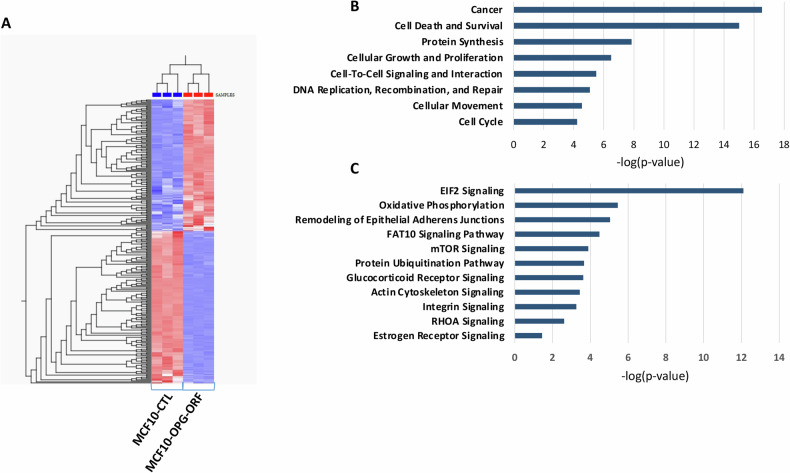
OPG upregulation affects the expression of several genes involved in various physiological processes. Protein extracts were prepared from MCF-10A cells expressing either an empty vector (MCF10-CTL) or a vector bearing OPG-ORF (MCF10-OPG-ORF), and were used for proteomics analysis. **A** Two-dimensional hierarchical clustering using significant proteins (DEP) separated MCF10-CTL from MCF10-OPG-ORF. Samples are denoted in columns and proteins are denoted in rows. **B** Overrepresented biological functions and (**C**) significantly altered canonical pathways associated with DEP (up- or downregulated). X-axis indicates the significance (−log *P* value).

Next, we sought to delineate the effect of OPG upregulation on protein expression in MCF-10A cells. To this end, whole cell lysates were prepared from MCF10-OPG-ORF and their control cells, and were then subjected to 2D proteomic analysis. The Hit map shows 274 differentially expressed proteins (DEPs) that were significantly dysregulated (*p* < 0.05, fold change >2) (Fig. 4A). DEPs were mapped to their respective gene symbols using UniProt and the IPA Knowledgebase. The corresponding genes (DEGs) were further investigated in terms of their functions and gene networks. Gene ontology and functional analyses revealed significant enrichment of genes that are involved in cancer, cell death, protein synthesis, cellular growth and proliferation, cell-to-cell signaling and interaction, DNA replication, recombination and repair, cellular movement, and cell cycle (Fig. 4B). The significantly altered canonical pathways include EIF2 signaling, oxidative phosphorylation, remodeling of epithelial adherent junctions, FAT10 signaling, and integrin signaling, among others (Fig. 4C). Gene interaction network analysis of DEGs indicated top significant subnetworks related to a number of cancer-related pathways and hub genes, including E2F, PI3K, p38 MAPK, JNK, Rb, CDK1, and MAP2K (Fig. S3).

### OPG expression correlates well with the EMT and stemness genes

**Fig. 5:**
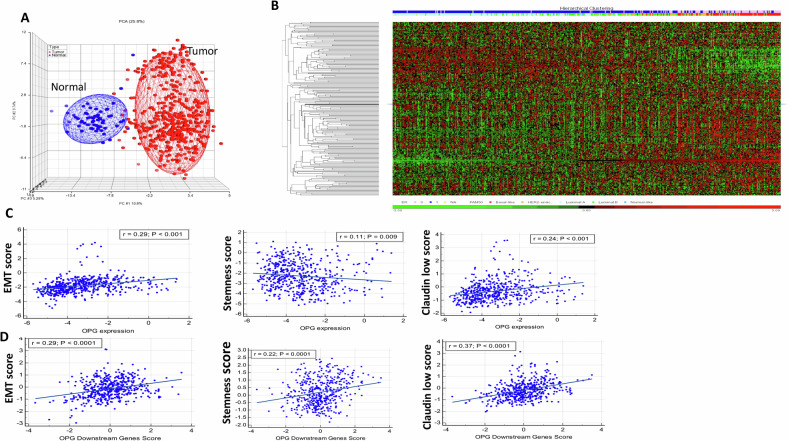
OPG expression correlates well with EMT, claudin low and stemness genes. **A** Unsupervised principal component analyses (PCA) using the TCGA dataset (breast invasive carcinoma samples (n = 460) and normal (n = 62)) clearly distinguished patients from normal samples. **B** The two-dimensional hierarchical clustering data from the invasive breast cancer patients. The gene cluster that included a high expression of OPG displays overrepresentation of samples from ER-negative and Basal-like subtype. The arrow indicates the *OPG* gene. The red and green colors in the heatmap indicate highly or weakly expressed genes, respectively. The pink and red bars above the heatmap represent ER and Basal-like subtypes, respectively (see Legend). **C**, **D** Correlation of OPG expression with EMT, stemness and claudin-low features using TCGA dataset. **C** EMT, Stemness, and Claudin Low Scores vs OPG (TNFRS11B) gene expression. **D** OPG-downstream genes score, that is based on the genes/proteins that are affected by the OPG expression vs EMT, Stemness, and Claudin Low Scores. Scores are calculated for each sample in the TCGA data, as described in the methods.

Next, we performed unsupervised principal component analyses (PCA) using the DEGs on the TCGA data, which clearly distinguished patients from normal samples (Fig. 5A). The two-dimensional hierarchical clustering of the TCGA data from patients with invasive breast cancer revealed that a gene cluster that included the *OPG* gene has an overrepresentation of samples from patients with ER and basal-like subtypes of cancer (Fig. 5B). We have next investigated the association between OPG expression and EMT/stemness status in breast cancer patients. For each sample in the TCGA data, we calculated an EMT score, stemness and Claudin-low (CL) scores as has been previously described [16–18] and searched for their correlation with OPG expression. Interestingly, the expression of the *OPG* gene and its downstream target genes were significantly correlated with EMT, stemness, and claudin-low scores (Fig. 5C, D). This indicates that OPG expression is associated with the features of cancer stem cells.

### OPG upregulation promotes the paracrine pro-carcinogenic signaling of breast cancer cells

**Fig. 6:**
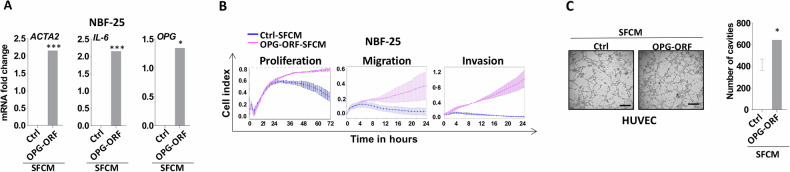
OPG upregulation in epithelial cells promotes their paracrine pro-carcinogenic effects. T-47D cells (Ctrl-ORF) were cultured in SFM for 24 h, and the respective SFCM (Ctrl-SFCM and OPG-ORF-SFCM) were collected (**A**) Total RNA was prepared from NBF-25 cells that were treated with either Ctrl-SFCM or ORF-SFCM for 24 h, and then the expression level of the indicated genes was assessed using qRT‐PCR. Error bars represent mean ± SEM (*n* = 3). **P ≤ 0.01; ***P ≤ 0.001. **B** Ctrl-SFCM and OPG-ORF-SFCM were used to treat NBF-25 cells for 24 h, and then cell proliferation, migration, and invasion abilities were determined using the real-time xCELLigence RTCA System. **C** HUVEC cells grown on matrigel (96‐well plate) were treated with Ctrl-SFCM or ORF-SFCM. The number of tube cavities was counted at ×10 magnification after 4 h of incubation. Representative photographs of HUVEC cavities (left panel), number of cavities (right panel). Scale bars represent 100 μm. Error bars represent mean ± SEM (*n* = 3). *P ≤ 0.05.

After showing the endogenous effect of OPG upregulation on breast epithelial/cancer cells, we sought to test the non-cell-autonomous effects of this upregulation on breast fibroblasts as well as endothelial cells. Therefore, OPG-expressing T-47D cells and their corresponding control cells were cultured in serum-free medium (SFM) for 24 h, and then serum-free conditioned media (SFCM) were collected (OPG-ORF-SFCM and Ctrl-SFCM, respectively). Next, SFCM were used to treat normal breast stromal fibroblasts (NBF‐25) for 24 h, and then total RNA was prepared for real-time RT-PCR amplification/analysis. Figure 6A shows that OPG-ORF-SFCM potently upregulated the levels of the three major markers of active fibroblasts: *ACTA2*, *CXCL12* and *IL‐6* in NBF‐25 cells as compared to those treated with Ctrl-SFCM. In addition, the mRNA level of OPG was increased in NBF-25 cells exposed to OPG-ORF-SFCM (Fig. 6A). This prompted us to functionally confirm these findings by examining the proliferation, migration, and invasion abilities of these NBF‐25 cells. Figure 6B shows that OPG-ORF-SFCM enhanced the proliferation, migration, and invasion abilities of NBF‐25 cells as compared to Ctrl-SFCM. These results reveal that OPG upregulation in breast cancer cells enhances the paracrine activation ability of normal breast stromal fibroblasts.

Next, we investigated the pro-angiogenic effects of OPG upregulation in breast epithelial cells in vitro. Therefore, HUVEC cells were cultured either with OPG-ORF-SFCM or Ctrl-SFCM in 96‐well plate pre‐coated with matrigel. Figure 6C shows that the number of formed close cavities was significantly higher in the presence of OPG-ORF-SFCM than in the presence of Ctrl-SFCM. This indicates that OPG upregulation in breast cancer cells enhances their paracrine promotion of endothelial cells differentiation.

### OPG downregulation suppresses the aggressive features of triple-negative BC cells

**Fig. 7:**
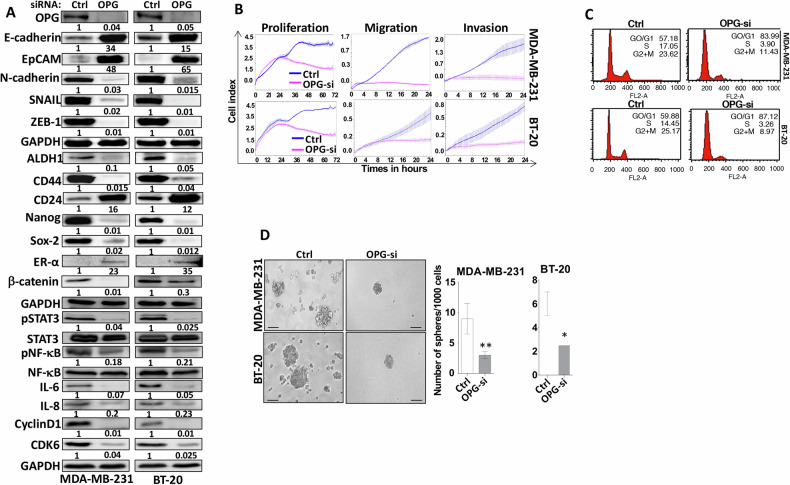
OPG downregulation inhibits the breast carcinogenesis of breast cancer cells. MDA-MB-231 and BT-20 cells were transfected with OPG siRNA (OPG-si), while a scrambled sequence was used as control (Ctrl). **A** Western blot images with GAPDH used as internal control. The numbers underneath each band represent fold changes relative to the control (Ctrl) after normalization to GAPDH. Phosphorylated protein fractions were determined by quantification and normalization against their relative non-phosphorylated forms (**B**) Cellular analysis using the real-time xCELLigence RTCA System. **C** Cells (2 × 10^5^) were harvested and stained with propidium iodide (PI), and were analyzed by flow cytometry. The fractions of cells in each phase of the cell cycle are depicted in the boxes. **D** Cells (1 × 10^3^) were seeded in ultra-low attachment 96‐well plate containing stem cell‐specific medium. The number of spheroids (>100 μm) were counted. Representative photographs of spheroids (left panel), number of formed spheroids (right panel). Error bars represent mean ± SEM (*n* = 3). *P ≤ 0.05; **P ≤ 0.01.

To further elucidate the role of OPG in breast carcinogenesis, OPG was knocked down using a specific OPG siRNA in the triple-negative breast cancer (TNBC) cells, namely MDA-MB-231 and BT-20, while a scrambled sequence was utilized as a control (OPG-si and Ctrl, respectively). Figure 7A shows that the level of the OPG protein was decreased in OPG-deficient cells compared with controls. This downregulation of OPG in breast cancer cells was accompanied with an increase in the expression of the ERα protein and the epithelial markers E-cadherin and EpCAM, while the mesenchymal markers N-cadherin, Snail, and ZEB1 were downregulated (Fig. 7A). In addition, Fig. 7B shows that OPG siRNA decreased the proliferation, migration, and invasion abilities of MDA-MB-231 and BT-20 cells as compared to their respective controls. Therefore, the downregulation of OPG suppressed the EMT process in TNBC cells and promoted their transition into the epithelial state. The anti-proliferative effect of OPG siRNA prompted us to test the effect of OPG downregulation on the cell cycle. To this end, exponentially growing OPG-deficient cells and their respective controls were stained with propidium iodide (PI), and their progression in the cell cycle was analyzed by flow cytometry. Figure 7C shows that OPG downregulation resulted in cellular accumulation at the G0/G1 phase of the cell cycle. Indeed, the proportion of cells in G0/G1 increased from 57.18% and 59.88% to 83.99% and 87.01% in MDA-MB-231 and BT-20 cells, respectively. To confirm this, the levels of Cyclin D1 and CDK6 proteins, two major regulators of the G1/S transition, were assessed by immunoblotting. Figure 7A shows that the levels of both proteins were clearly reduced in both breast cancer cell lines. These results indicate that the downregulation of OPG in TNBC cells inhibits their proliferative capacities through cell cycle arrest at the G0/G1 phase.

To confirm the role of OPG in promoting the carcinogenesis of BC cells and their stemness characteristics, we tested the effect of OPG knockdown on the stemness features of breast cancer cells. Figure 7A shows that OPG siRNA reduced the levels of the BCSC markers ALDH1, and CD44, and markedly increased the CD24 level in both cell lines. Likewise, Nanog, KLF4, Sox2, and β‐catenin were all downregulated in OPG knockdown cells compared with their respective controls (Fig. 7A). These molecular results on stemness biomarkers were confirmed at the cellular level by in vitro spheroid formation assay, which showed that OPG siRNA significantly decreased the number of formed spheroids in MDA-MB-231 and BT-20 cells compared with their respective controls (Fig. 7D). These results indicate that the downregulation of OPG reduced the stemness features of breast cancer cells. Moreover, OPG siRNA inhibited the STAT3 and NF-κB transcription factors and suppressed the expression of their downstream mediators IL-6 and IL-8 in both MDA-MB-231 and BT-20 cells (Fig. 7A). These results indicate that the downregulation of OPG in TNBC cells upregulated ERα and inhibits its aggressive features: proliferation, migration/invasion, and stemness.

### OPG level controls the sensitivity of TNBC cells to chemotherapeutic drugs

**Fig. 8:**
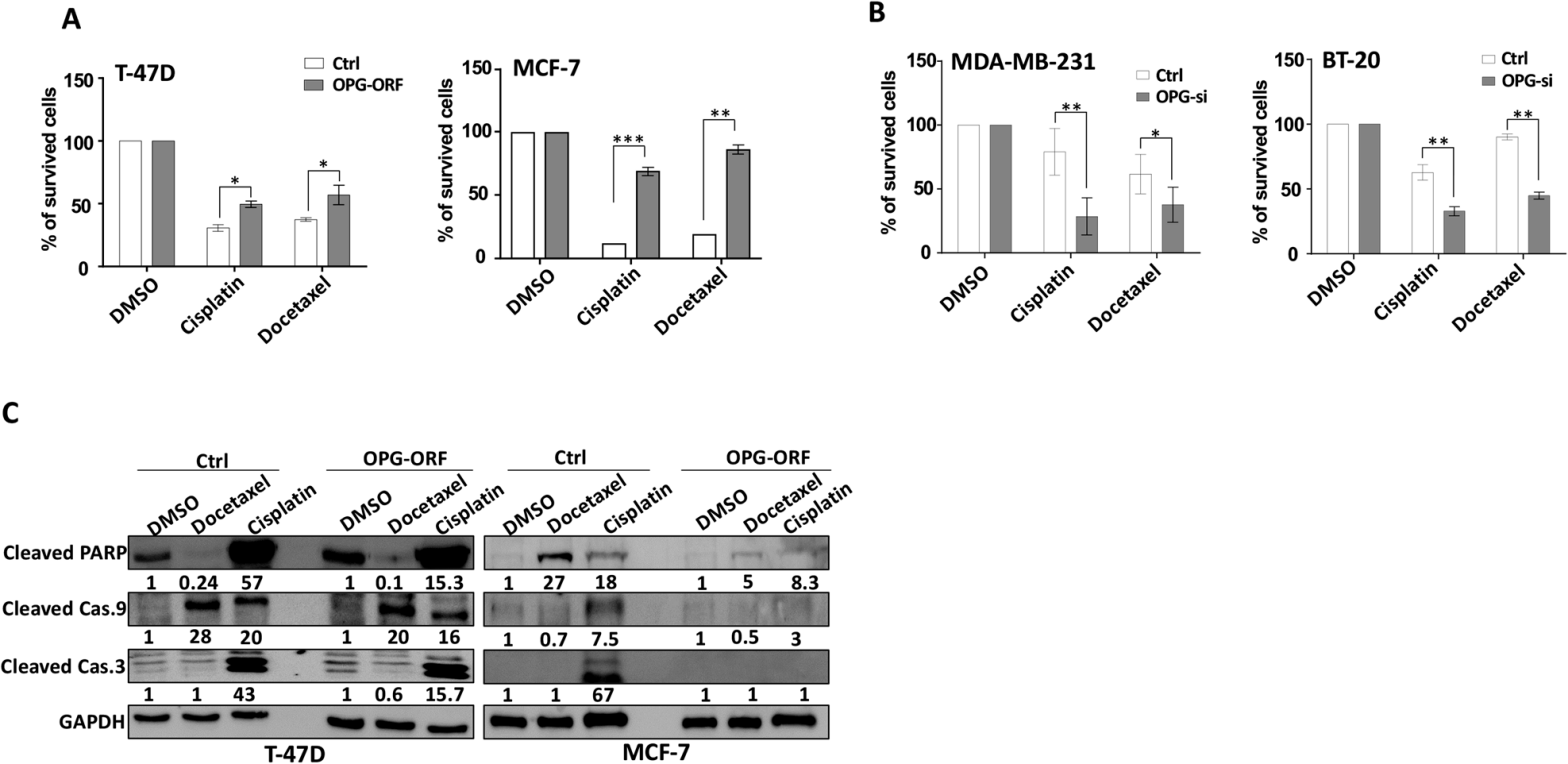
OPG-dependent promotion of BCSCs induces resistance of breast cancer cells to chemotherapeutic drugs. **A**, **B** The indicated cells (5 × 10^3^ cells per well) were seeded in 96‐well plates, and then were either sham-treated or challenged with cisplatin (50 and 100 μM) or docetaxel (5 μg/ml) for 72 h. Cell cytotoxicity was assessed using the WST1 assay and recorded as percent of survived cells relative to the untreated cells. Error bars indicate mean ± SEM (*n* = 3). *P ≤ 0.05, **P ≤ 0.01, ***P ≤ 0.001. **C** Western blot images with GAPDH used as internal control. The numbers underneath each band represent fold changes relative to the control (Ctrl) after normalization to GAPDH.

Because OPG promotes BCSCs, which enhance resistance of breast cancer cells to chemotherapy, we sought to test the effect of OPG expression on the sensitivity of breast cancer cells to widely used chemotherapeutic drugs. To this end, T-47D and MCF-7 cells expressing either OPG-ORF or an empty vector were either sham-treated or challenged with cisplatin (100 μM) or docetaxel (5 μg/ml) for 72 h. The WST1 assay was performed to evaluate the response of cells to both drugs. Figure 8A shows that the upregulation of OPG in breast cancer cells increased their resistance to both cisplatin and docetaxel compared with their respective control cells. Indeed, OPG-expressing cells showed twofold (T-47D) and 6-fold (MCF-7) higher resistance in response to both chemotherapeutic drugs (Fig. 8A). To confirm this finding at the molecular level, we evaluated the effect of cisplatin and docetaxel on the level of the important pro-apoptotic proteins PARP, caspase-9, and caspase-3 in both T-47D and MCF-7 cells by immunoblotting. The obtained results indicate that OPG upregulation reduced the cisplatin- and docetaxel-dependent increase in the levels of cleaved-PARP, cleaved-caspase-3 and cleaved-caspase-9 compared with their respective controls in both cell lines (Fig. 8B). To further confirm the OPG-dependent increase in cellular resistance to chemotherapy, we tested the effect of OPG downregulation on the response of breast cancer cells to both cisplatin and docetaxel. To this end, OPG-deficient MDA-MB-231 and BT-20 cells and their respective controls were either sham-treated or challenged with cisplatin (50 μM) or docetaxel (5 μg/ml) for 72 h. Figure 8D shows that the downregulation of OPG in breast cancer cells caused a significant decrease in their resistance to both cisplatin and docetaxel compared with their respective controls. These results show that the OPG-dependent promotion of BCSCs enhances the resistance of breast cancer cells to chemotherapeutic drugs.

## Discussion

OPG is a multifunctional secreted cytokine with both pro- and anti-carcinogenic effects [9]. In the present report, we have shown that OPG is a major regulator of stemness in mammary epithelial cells. Indeed, OPG upregulation promoted the pro-metastatic processes EMT and stemness in the non-carcinogenic (MCF-10A) and luminal breast cancer (T-47D, MCF-7) cells, through upregulating the mesenchymal (N-cadherin, SNAIL and ZEB1) and stemness (CD44, ALDH1, Nanog, Klf-4 and Sox2) markers. These effects were mediated through different pathways (STAT3, NF-κB and β-catenin), which enhanced the proliferative, migratory/invasive and self-renewal capacities of epithelial cells in vitro and tumor growth in vivo. Similarly, OPG overexpression in breast cancer cells enhances proliferation and orthotopic tumor growth [19, 20].

The OPG-dependent changes in gene expression could result from the activation of NF-κB, an important target of the OPG/RANKL pathway [9, 21]. Indeed, OPG upregulation activated NF-κB and its downstream target genes IL-6 and IL-8 as well as the related transcription factor STAT3. The other important CSC-related pathway is the WNT/β-catenin signaling, which is activated in 50% of breast cancer patients and is associated with poor survival [22]. Accordingly, we have shown here that OPG upregulation activates β-catenin, promotes its accumulation into the nucleus and increases the expression of its downstream target genes *CD44*, *AXIN2*, *c-MYC*, *IL-6*, and *ALDH1A1*. On the other hand, OPG-specific knockdown reduced the level of the β-catenin protein in two aggressive TNBC cell lines (MDA-MB-231 and BT-20). This shows that OPG upregulation can promote stemness in breast epithelial cells through either the STAT3 or the β-catenin signaling pathways. Likewise, we have previously shown that OPG upregulation activates breast stromal fibroblasts and enhances their pro-carcinogenic effects in a STAT3-dependent manner [23]. This shows that endogenous OPG can enhance pro-carcinogenic features in both cancer and stromal cells by activating the STAT3/IL-6 pathway. This was confirmed by showing that OPG upregulation affected the expression of several proteins, and the OPG levels correlated with the expression of proteins with functions in EMT and stemness. This confirmed a direct link between OPG expression levels and the promotion of stemness in breast epithelial cells. This explains the detection of high levels of OPG in human breast cancer tissue samples compared with the uninvolved tissues from the same patients [19]. In addition, OPG was found to be expressed in 45% [24] and 55% [25] of breast cancer tissues, and 40% of invasive breast tumors showed a strong immunohistochemical expression of OPG [10]. Furthermore, OPG has metastasis-promoting effects in triple-negative breast cancer cells [15]. Together, these findings demonstrate the importance of OPG upregulation for breast carcinogenesis. Furthermore, the fact that OPG controls these 3 important signaling pathways, namely NF-κB, STAT3 and WNT/β-catenin explains the implication of OPG in the various autocrine and paracrine physiological processes such as cell cycle and angiogenesis [9].

The other major factor in breast carcinogenesis is ERα, which is also a transcription factor and an RNA-binding protein that regulates the expression of a plethora of genes involved in cell cycle, proliferation, and other important cancer-related physiological processes [26]. We have shown here that OPG negatively regulates ERα. Indeed, while OPG upregulation decreased the expression of ERα in breast cancer luminal cells (T-47D) and the non-cancerous MCF-10A cells, OPG knockdown upregulated ERα in the TNBC cells. Furthermore, using 2D proteomic analysis, we have shown that OPG upregulation correlates well with the expression of genes related to the ER-negative claudin low subtype of TNBC cells. Importantly, the two-dimensional hierarchical clustering of the TCGA data from invasive breast cancer patients revealed that a gene cluster that includes the OPG gene has overrepresentation of samples from patients with ER-negative and basal-like subtypes of breast cancer. Similarly, a strong negative correlation was found between the expression of OPG and ERα in endothelial cells [27]. These findings raised an important question on how OPG represses the expression of ERα. This effect could me mediated through the transcription factor NF-κB, which is a target of OPG (Figs. 1B and 7A). Indeed, Wang et al. have previously shown that NF-κB (RelB) represses the expression of ERα via induction of the zinc finger protein Blimp1 [28, 29].

The pro-carcinogenic effects of OPG upregulation in breast epithelial cells were further confirmed by showing that these cells can activate breast fibroblasts and enhance the differentiation of endothelial cells into cavities in a non-cell-autonomous manner. Similarly, it has been previously shown that OPG may be involved in the regulation of endothelial cell phenotype and tumor angiogenesis [27].

In addition, we have shown that OPG downregulation inhibits the proliferation of TNBC cells through cell cycle arrest at G0/G1 phase. This was confirmed by showing a strong decrease in the levels of the 2 main cell cycle regulators cyclin D1 and CDK6. This suggests a potential role of OPG in the G1-S transition. In fact, it has been recently shown that OPG supplementation improved the proliferation of islet β cells in a rat model of intrauterine growth retardation (IUGR) [30]. This confirms that OPG functions are not limited to the osteoclast type of cells and bone metabolism. OPG downregulation also inhibited the NF-κB/STAT3 signaling pathways, EMT, and stemness processes in TNBC cells, confirming the role of OPG in these pro-matastatic processes. Indeed, it was previously shown that OPG knockdown in MDA-MB-231 cells reduces their metastasis in a chick embryo metastasis model [15]. OPG-dependent promotion of chemoresistant cancer stem cells was confirmed by showing that while OPG upregulation enhanced the resistance of BC cells to cisplatin and docetaxel, OPG downregulation enhanced the sensitivity of TNBC cells to these chemotherapeutic drugs (Fig. 8).

BCSCs survive various types of chemotherapeutic agents owing to their cell dormancy, higher DNA repair abilities and drug efflux [31]. In the present study, we have shown that OPG upregulation increases the expression of ALDH1, a detoxification enzyme that metabolizes anti-cancer molecules and increases the mitochondrial quality [32]. These findings suggest that high expression level of OPG in breast cancer tissues should be associated with poor survival of BC patients. However, the data reported so far are puzzling, with OPG expression linked to both good and poor prognosis [33].

This indicates that targeting endogenous OPG signaling could be of great therapeutic value for a good proportion of BC patients, through targeting the cancer stem cell subpopulation. These cells are responsible for the progression, recurrence, and the resistance of these neoplasms [31].

Like OPG, denosumab is also an inhibitor of the interaction of RANKL with its receptor RANK, which activates the NF-κB pathway [34, 35]. We have recently shown that denosumab can target CSCs and inhibit various relevant CSC-related pathways such as the NF-κB and β-catenin signalings [36]. Denosumab is used to treat breast cancer patients at high risk of bone fractures, and also those suffering from bone metastasis [35]. Denosumab may also be useful for the treatment of patients with early breast cancer in adjuvant and/or neoadjuvent settings. In fact, these possibilities have been tested in several clinical trials [9, 37, 38]. This indicates that denosumab could improve the neoadjuvent treatment of certain breast cancer patients through potentiating the anti-CSC effect of some chemotherapeutic drugs. Another possibility is to use recombinant human OPG (rhOPG), which has been shown to downregulate endogeneous OPG in breast cancer cells and target the CSC subpopulation [36]. In fact, recombinant truncated OPG (AMGN-0007) can suppress bone resorption in patients with multiple myeloma or breast carcinoma [39]. Moreover, we have recently shown that rhOPG can inhibit the growth of humanized orthotopic TNBC tumor xenografts in nude mice [36]. Thereby, in vitro and in vivo investigations are still needed to delineate the use of rhOPG/denosumab for the treatment of BC patients. Despite the fact that targeting the OPG/RANK/RANKL pathway is a promising therapeutic strategy for breast tumors, this approach seems to be limited by the critical role of this pathway in bone remodeling, and other physiological processes. Therefore, future studies are required in order to further understand the role of OPG in breast carcinogenesis/metastasis and osteolysis and the potential link between these processes.

## Conclusions

Together, these data shed more light on the role of OPG in breast carcinogenesis. Indeed, we have shown that OPG is a major player in cell cycle, EMT, stemness and angiogenesis processes through the regulation of various key signaling pathways. This indicates that OPG could be of great value for precision therapy of breast cancer patients. Thereby, identification, and in vitro and in vivo characterization of potent inhibitors of the OPG/RANK/RANKL pathway, will pave the way toward specific targeting of the chemoresistant cancer stem cells.

## Materials and methods

### Cells, cell culture, and reagents

Breast fibroblast cells (NBF‐25) were obtained and used as previously described [40]. MCF-10A, MCF-12A, MCF-7, T-47D, MDA-MB-231, MDA-MB-468, SKBR-3, BT-474, BT-20 and human umbilical vein endothelial cells (HUVEC) cell lines were purchased from ATCC, SUM149PT cell line was purchased from Asterand Bioscience. All cells were authenticated using short tandem repeat profiling, propagated, expanded, and frozen immediately into numerous aliquots after arrival. The revived cells were utilized within 10 to 12 passages and not exceeding a period of 3 months. Cells were regularly screened for mycoplasma contamination using Mycoplasma Detection Kit (ATCC). Cells were cultured as recommended and were grown at 37 °C in an incubator (humidified, 5% CO2). MCF-10A and MCF-12A were cultured in DMEM/F12 medium supplemented with 10% Fetal Bovine Serum (FBS), 1% Antibiotic-Antimycotic (AB), 1% HuMEC and 0.5% Bovine Pitutary Extract. MCF-7, T-47D, MDA-MB-231, MDA-MB-468, and HUVEC were cultured in RPMI medium supplemented with 10% FBS and 1% AB. SKBR-3 and BT-474 were cultured in McCoy′s 5 A medium supplemented with 10% FBS and 1% AB. BT-20 was cultured in DMEM/F12 medium supplemented with 10% FBS and 1% AB. SUM149PT was cultured in RPMI medium supplemented with 10% FBS and 1% AB, 5 µg/ml insulin and 1 µg/ml hydrocortisone. All supplements were obtained from Gibco except insulin and hydrocortisone from Sigma-Aldrich. Cisplatin was purchased from Sigma-Aldrich. Docetaxel was purchased from Pfizer.

### Transfections

OPG-ORF and its corresponding control (1 µg) (Origene) were used to transfect cells using Lipofectamine 3000 following the manufacturer’s instructions (Invitrogen). As negative control cells were transfected in the absence of plasmid. Transfected cells were selected using puromycin (Gibco), until all negative control cells died. The transfection efficiency reached 20%.

Transfection with OPG siRNA and universal scrambled sequence (30 nM) (Santa Cruz Biotechnology) was performed using the RNAiFect reagent (Qiagen) following the manufacturer recommendations.

### RNA purification and qRT-PCR

mRNeasy mini kit (Qiagen) was used to purify total RNA as recommended by the manufacturer’s instructions. RNA concentration and integrity were determined using Agilent 2100 bioanalyzer system and associated Agilent 2100 expert software. RNA (1 µg) was used to synthesize complementary deoxyribonucleic acid (cDNA) using the Advantage RT-PCR kit (Clontech Laboratories) following the manufacturer’s instructions. Amplifications were performed using FastStart Essential DNA Green Master (Roche) and were performed using the LightCycler® 96 Real‐time PCR detection system (Roche) according to the following cycle conditions: 95 °C for 10 min (1 cycle); 95 °C for 10 s, 59 °C for 20 s, and 72 °C for 30 s (45 cycles). GAPDH was used for normalization, and gene expression differences were calculated using the threshold cycle (Ct) by LightCycler® 96 SW 1.1 software. Primer sequences are shown in Table S1. These experiments were performed in triplicate and repeated 3 times.

### Cellular lysate preparation and immunoblotting

Cells were harvested, centrifuged, and the pellets were homogenized with RIPA buffer (Sigma-Aldrich) supplemented with protease inhibitors (Roche). Cells were incubated on ice with a brief vortex every 10 min for 2 h. The obtained cell lysates were centrifuged, and the resulting supernatants were stored at −80 °C. Extracted proteins (50 μg) were separated using SDS-PAGE, and then were transferred to polyvinylidene difluoride membrane (PVDF) (Bio-Rad), which was first blocked with 5% powdered skimmed milk in Tris buffered saline with tween (TBST) (Sigma-Aldrich) for 1 h, and then was incubated with the appropriate primary antibody (diluted in TBST as recommended by the suppliers) overnight, and then with an appropriate secondary antibody (Promega) for 1 h. As negative controls membranes were stained only with secondary antibodies. Visualization of the secondary antibody was performed using a chemiluminescence detection procedure according to the manufacturer’s protocol (Thermo Fisher Scientific).

Primary antibodies directed against IL-6 [1.2-2B11-2G10] (cat#ab9324), IL-8 [EP1117Y] (cat#ab52612) and ZEB1 [2A8A6] (cat#ab181451) were purchased from Abcam. OPG [N-20] (cat#sc-8468), ERα [10H12B10] (cat#sc-130072), CDK6 [C-21] (cat#sc-177), CD24 [SN3] (cat#sc-19585) and GAPDH [FL-335] (cat#sc-25778) were purchased from Santa Cruz Biotechnology. E-cadherin [24E10] (cat#3195), N-Cadherin [13A9] (cat#14215), NF-κB p65 [D14E12] (cat# 8242), pNF-κB p65 (Ser536) (cat#3031), Nanog [D73G4] (cat#4903), Oct-4 [C30A3] (cat#2840), KLF4 [D1F2] (cat#12173), Sox2 [D6D9] (cat# 3579), Snail [C15D3] (cat# 3879), Bmi1 [D20B7] (cat#6964) STAT3 [124H6] (cat#9139S), p-STAT3 (Tyr705) [D3A7] (cat#9145), cyclin D1 (cat#2922), β−catenin [6B3] (cat#9582S), α-tubulin [11H10] (cat#2125), cleaved-PARP [Asp214] (cat#9541), cleaved caspase-9 [Asp315] (cat#9505), cleaved caspase-3 [Asp175] (cat# 9664) and EpCAM [D1B3] (cat#2626) were purchased from Cell Signaling Technology. CD44 was purchased from Sigma-Aldrich (cat# HPA005785). ALDH1 was purchased from BD Biosciences (cat#611195). These experiments were repeated 3 times.

### Immunofluorescence

Cells (2 × 10^5^) were fixed in formaldehyde (4%) for 19 min and blocked with goat serum (5%), triton ×-100 (0.3%), and sodium azide (0.05%) for 1 h. The slides were then stained overnight at 4 °C with OPG (cat#sc-8468) (Santa Cruz Biotechnology) and ALDH (cat#611195) (BD Biosciences) antibodies, diluted as recommended by the respective suppliers in BSA (1%), triton × (0.3%) and sodium azide (0.05%). Subsequently incubated with Alexa flour 594-conjugated goat anti-mouse IgG (Thermo Fisher Scientific), Rhodamine-conjugated rabbit anti-goat IgG Antibody (Sigma-Aldrich) and DAPI (Thermo Fisher Scientific) for 1 h. As negative controls, slides were stained only with secondary antibodies. Images were acquired using a fluorescence microscope and ZEN Microscopy Software (ZEISS). These experiments were repeated 3 times.

### Cytotoxicity assay

Cells (5 × 10^3^) were first seeded in 96‐well plates with 100 µl appropriate complete culture medium, and incubated for 24 h to allow cells to attach. Cells were then treated for 72 h, then WST1 (10 µl) reagent (Roche) was added to each well according to the manufacturer’s instructions. After 4 h of the recommended incubation time, the absorbance of each sample was measured using a microplate (ELISA) reader at 450 nm (Bio-Rad). The absorbance value of the blank wells (containing only medium and WST1 reagent) was subtracted from the absorbance of each sample, then the values of treated samples were normalized to the controls. Control samples were represented as 100% cell viability and the percentage of viable cells in treated samples were calculated relative to the controls. These experiments were performed in triplicate and repeated 3 times.

### Cell proliferation, migration, and invasion assays

These assays were carried out using xCELLigence Real-Time Cell Analysis (RTCA) (Agilent technologies) as recommended by the manufacturer. For cell migration and invasion, (5 × 10^3^-2 × 10^4^) of exponentially growing cells in complete medium were harvested, and then seeded with serum-free medium (SFM) in the upper chamber wells of the CIM-plate non-coated (migration) or pre-coated (invasion) with a thin layer of matrigel (1:40) (BD Biosciences). Serum-containing medium 10% was used as a chemoattractant in the lower chamber wells. The plate was incubated in the RTCA system for 24 h. For the proliferation assay, cells (5 × 10^3^-2 × 10^4^) in complete medium were seeded in E-plates, and then incubated for 72 h. The RTCA pro software was used to analyze the obtained results expressed as Cell Index (CI) values. These experiments were performed in triplicate and were repeated at least 3 times.

### Flow cytometry

Cells (2 × 10^5^) were collected, washed, and incubated with CD44 APC-Cy7 (cat#559942) and CD24 PE (cat#555428) antibodies (BD biosciences) for surface staining (30 min on ice). Control cells were stained with isotype control, CD44 APC-Cy7 or CD24 PE alone. Data were acquired using the NovoCyte flow cytometer and NovoExpress software (Agilent technologies). Positive staining was considered on the basis of the negativity of an isotype control. A minimum of 10,000 events were recorded for all samples. Quality Control (QC) test was performed to monitor the performance of the NovoCyte flow cytometer before running the experiment using QC particles, which contain 5 kinds of microspheres with different fluorescence intensities and one blank microsphere. These experiments were repeated 3 times.

### Cell cycle analysis using flow cytometry

Propidium iodide (PI) stained cells (2 × 10^5^) were harvested, and then were analyzed by flow cytometry using the BD FACSCalibur™ flow cytometer, and the Cell Quest™ Pro operating software (BD biosciences). These experiments were repeated 3 times.

### Spheroid formation

Cells (1 × 10^3^) were seeded in 96-well ultra-low attachment plates in the presence of stem cell-specific medium (DMEM/F12 medium supplemented with 1% AB, 2% B-27, 20 ng/mL EGF, 20 ng/ml bFGF, 500 ng/ml hydrocortisone, 2 U/ml Heparin, 4% FBS and 5 μg/ml insulin). Cells were incubated for 10 days at 37 °C under 5% CO_2._ The aggregated clumps were dissociated by pipetting just before counting the spheroids. Spheroids with diameter of ≥100 μm were counted and photographed using Floid™ Cell Imaging Station (Life technologies). These experiments were repeated 3 times, and spheroid formation was consistent across replicates.

### Soft agar colony formation assay

Cells (4 × 10^4^) were washed, and then were resuspended in 4 ml of medium-containing agarose (0.3%) (Sigma-Aldrich). Single cells with agarose were plated in a 6-well plate with a layer of agarose (0.5%, v/v), and then were incubated at 37 °C under 5% CO_2_. The medium was changed every other day for 10 days. The formed colonies with a diameter of ≥100 μm were counted and photographed using an inverted light microscope (OLYMPUS). These experiments were repeated 3 times.

### Serum-free conditioned media

Cells were cultured in SFM for 24 h, and the media were collected, briefly centrifuged, and then were filtered. The resulting serum-free conditioned media (SFCM) were either used immediately or were frozen at −80 °C until needed.

### In vitro angiogenesis assay

HUVEC cells (2 × 10^4^) were mixed with SFCM, and then were seeded into 96-well plates pre-coated with matrigel (BD Biosciences) diluted in SFM (9:1) for 1 h. After incubation at 37 °C, the formed cavities were counted and photographed using an inverted light microscope (OLYMPUS). These experiments were repeated at least 3 times.

### Orthotopic tumor xenografts

Animal experiments were approved by the KFSH&RC institutional Animal Care and Use Committee (ACUC) under RAC#2180018, and were conducted according to relevant national and international guidelines. Different cell concentrations of T-47D-ORF and control T-47D-C (2 × 10^3^, 2 × 10^4^, 2 × 10^5^, 2 × 10^6^) cells (*n* = 3 for each cell concentration) were injected under the right and left nipples of female nude mice (Mus musculus, Nu/J, 6 weeks) respectively. After tumor growth, tumors were photographed. MCF-7-ORF or MCF-7-C (5 × 10^6^) cells (*n* = 5 each) were injected under the right nipples of female nude mice. After 1 month, the grown tumors were photographed. Animals were not randomized, and only suffered needle injection and tumor growth up to less than 2 cm, which was not exceeded. The experiments were performed with no blinding.

### Proteomics analysis using label-free quantitative liquid chromatography mass spectrometry (LC/MS)

Equal amounts (100 µg) of whole cell lysates were subjected to tryptic digestion as previously described [41, 42]. The resulting peptides were analyzed by mass spectrometry using label-free quantitative Nano Acquity liquid chromatography coupled to Synapt G2 by ion mobility separation HDMS^E^ on a Trizaic Nano-flow source (Waters). The MS data were acquired in the range of *m*/*z* 50-2000 Da for a 120 min gradient acquisition run time using ion mobility separation experiments (HDMS^E^). Each sample was analyzed in triplicate using the Mass Lynx platform (Version. 4·1, SCN833).

### Bioinformatics and data analysis

Progenesis QI was used for proteomics (QIfP) V 3·0 (Waters/Nonlinear Dynamics) for all automated data processing and database searching. Differentially expressed global proteins between samples were identified as previously described [41, 43]. Only proteins that are markedly different statistically (ANOVA), (*P* ≤ 0·05) with at least a 2 -fold change in their expression level were considered.

### Global gene expression analysis

Dataset from The Cancer Genome Atlas (TCGA) project (http://cancergenome.nih.gov) [44], which included mRNA expression profiling of breast invasive carcinoma samples (*n* = 460) and normal (*n* = 62) was used. The most highly processed data (TCGA level 3) were downloaded in accordance with the TCGA Data Access Policies (https://tcga-data.nci.nih.gov/tcga/). For each sample, the EMT score, stemness, and claudin-low (CL) scores were calculated as described previously [16–18]. Unsupervised principal component analysis (PCA) and two-dimensional hierarchical clustering using PARTEK Genomics Suite (Partek Inc.) were performed. Correlations between continuous variables were estimated using Pearson’s correlation coefficient (r), and associated *P* values were computed as previously described [16–18]. A *p* value of < 0.05 was considered significant.

### Functional pathway, and network analyses

Ingenuity Pathways Analysis (IPA) (QIAGEN, https://www.qiagenbioinformatics.com/products/ingenuity-pathway-analysis) was used for functional, and canonical pathway and protein-protein interaction network analyses. Differentially expressed proteins (DEPs) were mapped to their corresponding objects in the Ingenuity pathway knowledge base and gene interaction networks. A right-tailed Fisher’s exact test was used to calculate *p* values. All statistical tests were two-sided and *p*-value < 0.05 was considered statistically significant.

### Statistical analysis

Statistical analysis was performed using a two-tailed unpaired student’s *t*-test. *P* values of 0.05 and less were considered statistically significant. These analyses were performed using the GraphPad prism software. Statistical analysis related to proteomics and bioinformatics are described in the corresponding paragraphs.

### Supplementary information

Supplemental material

Supplementary information

## Data Availability

The data that support the findings of this study are available in the main figures and the supplementary material of this article, or from the corresponding author on reasonable request.
