# Supplementary material for: Endogenous osteoprotegerin (OPG) represses ERα and promotes stemness and chemoresistance in breast cancer cells
Source: Cell Death Discov. 2024 Aug 24;10:377. doi: 10.1038/s41420-024-02151-8 (PMC11344809; doi:10.1038/s41420-024-02151-8)
Supplement: Supplementary file 1 — Supplemental material [file 41420_2024_2151_MOESM1_ESM.pdf]

Figure 1

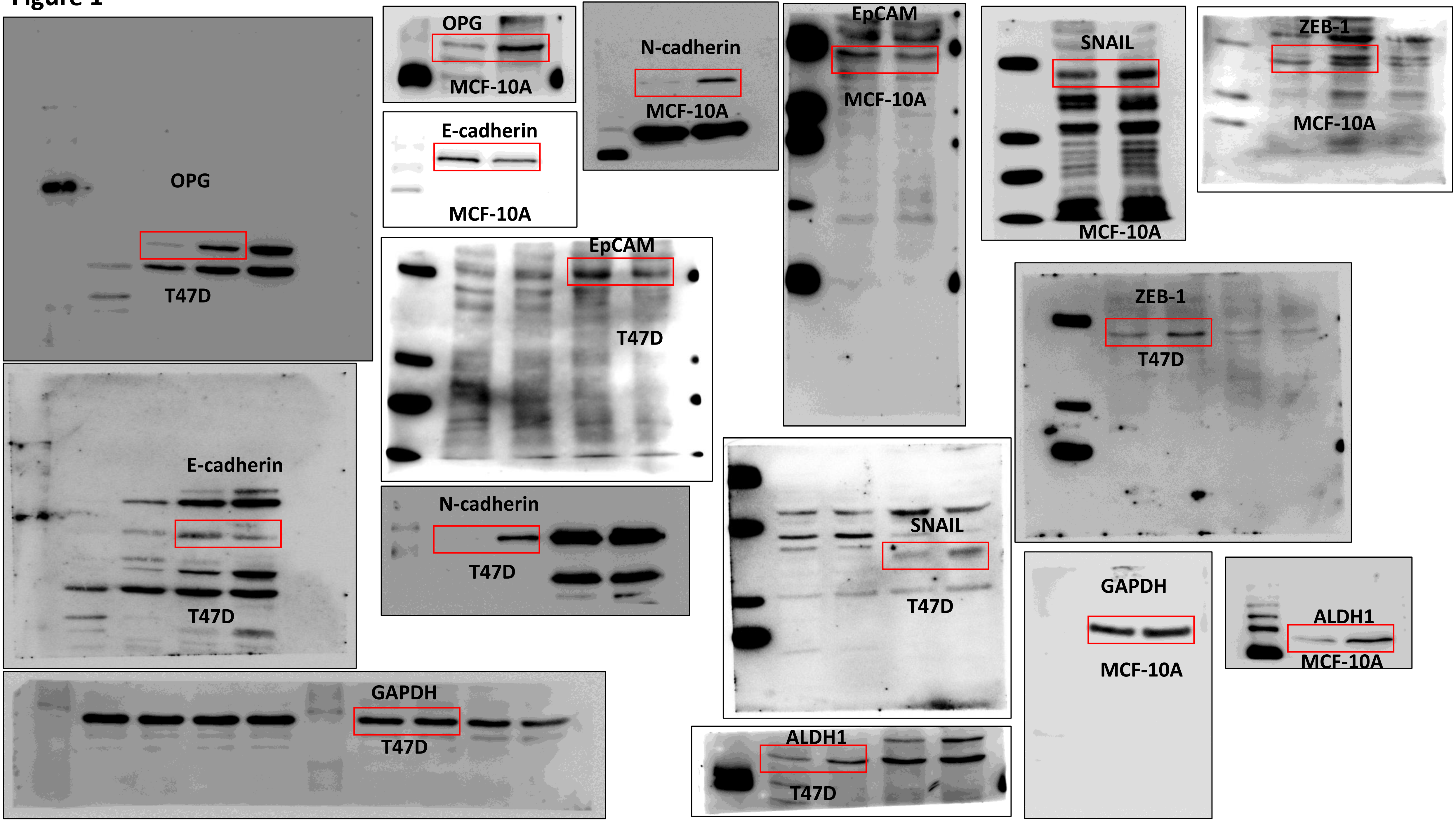
OPG
T47D
OPG
MCF-10A
E-cadherin
MCF-10A
N-cadherin
MCF-10A
EpCAM
MCF-10A
SNAIL
MCF-10A
ZEB-1
MCF-10A
EpCAM
T47D
ZEB-1
T47D
E-cadherin
T47D
N-cadherin
T47D
SNAIL
T47D
GAPDH
MCF-10A
ALDH1
MCF-10A
GAPDH
T47D
ALDH1
T47D

Figure 1

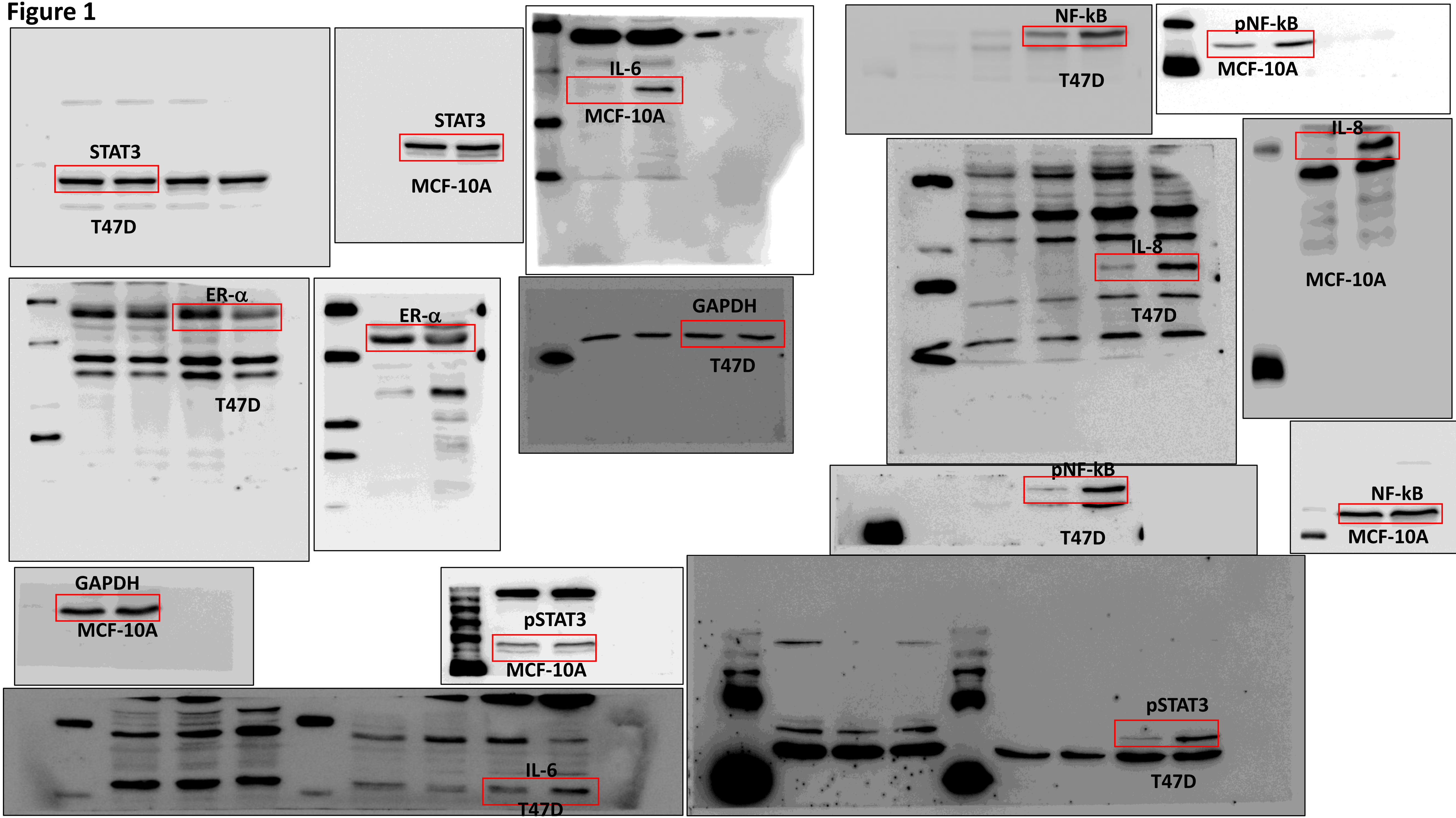
STAT3
T47D
STAT3
MCF-10A
IL-6
MCF-10A
NF-kB
T47D
pNF-kB
MCF-10A
IL-8
MCF-10A
IL-8
T47D
ER-α
T47D
ER-α
GAPDH
T47D
pNF-kB
T47D
NF-kB
MCF-10A
GAPDH
MCF-10A
pSTAT3
MCF-10A
IL-6
T47D
pSTAT3
T47D

Figure 2

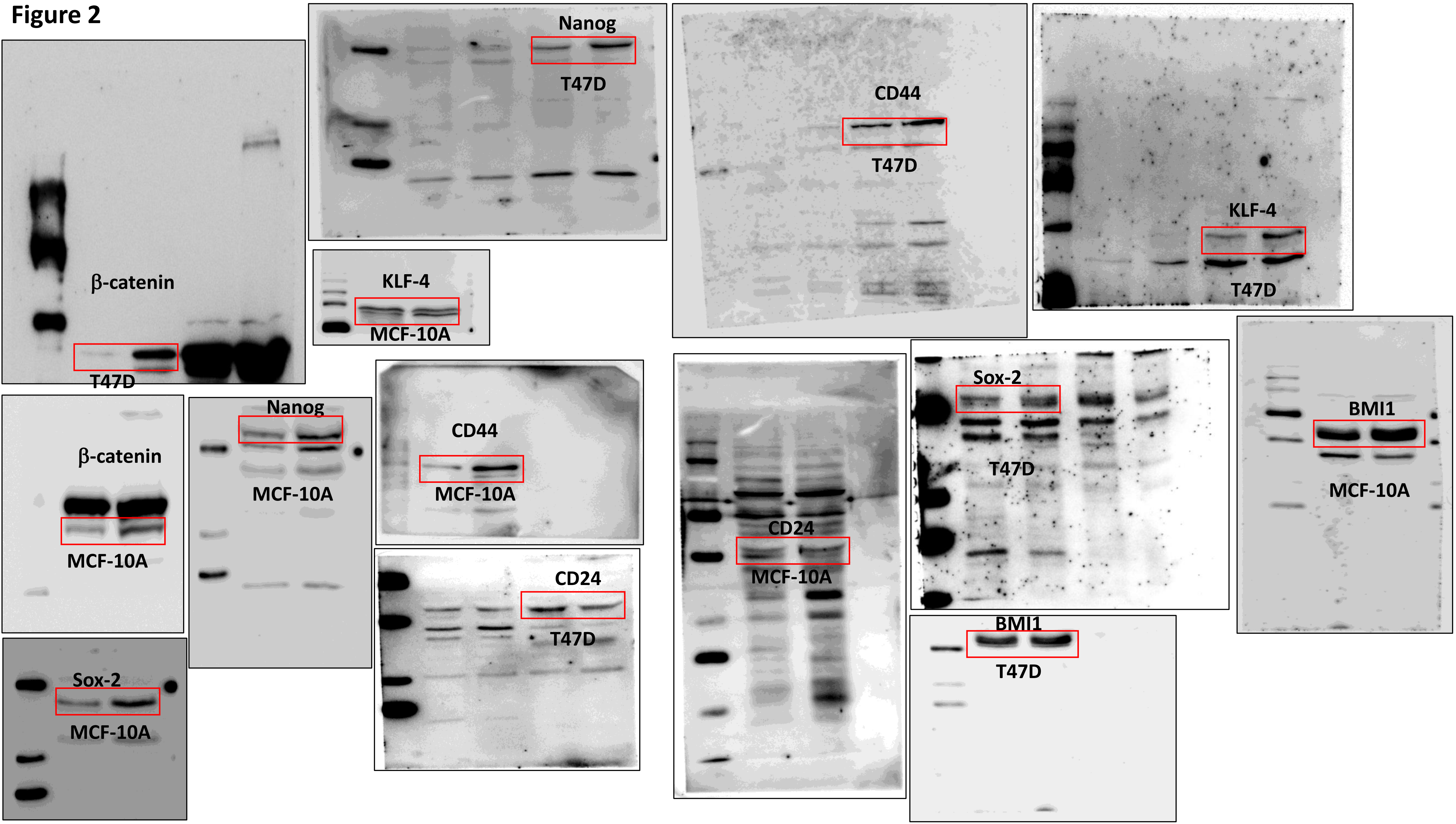

β-catenin
T47D
Nanog
T47D
KLF-4
MCF-10A
CD44
T47D
KLF-4
T47D
β-catenin
MCF-10A
Nanog
MCF-10A
CD44
MCF-10A
CD24
MCF-10A
Sox-2
T47D
BMI1
MCF-10A
CD24
T47D
BMI1
T47D
Sox-2
MCF-10A

Figure 2

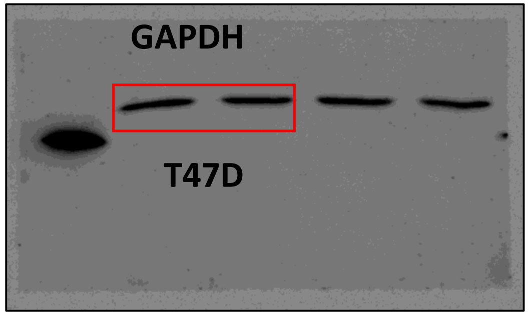

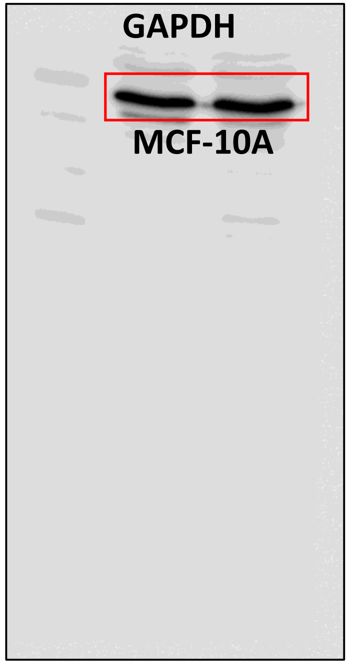

Supplementary Figure 1

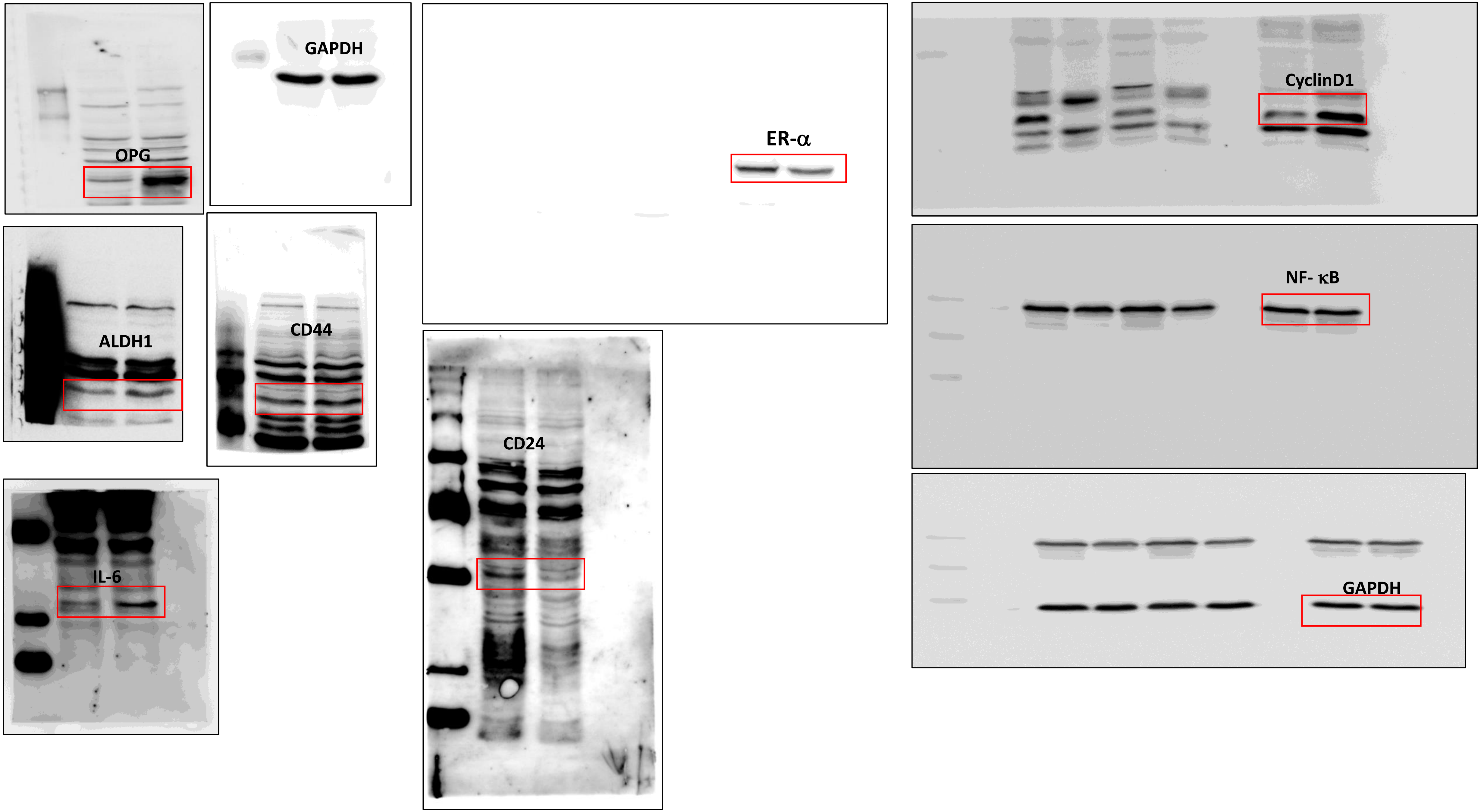

Supplementary Figure 1

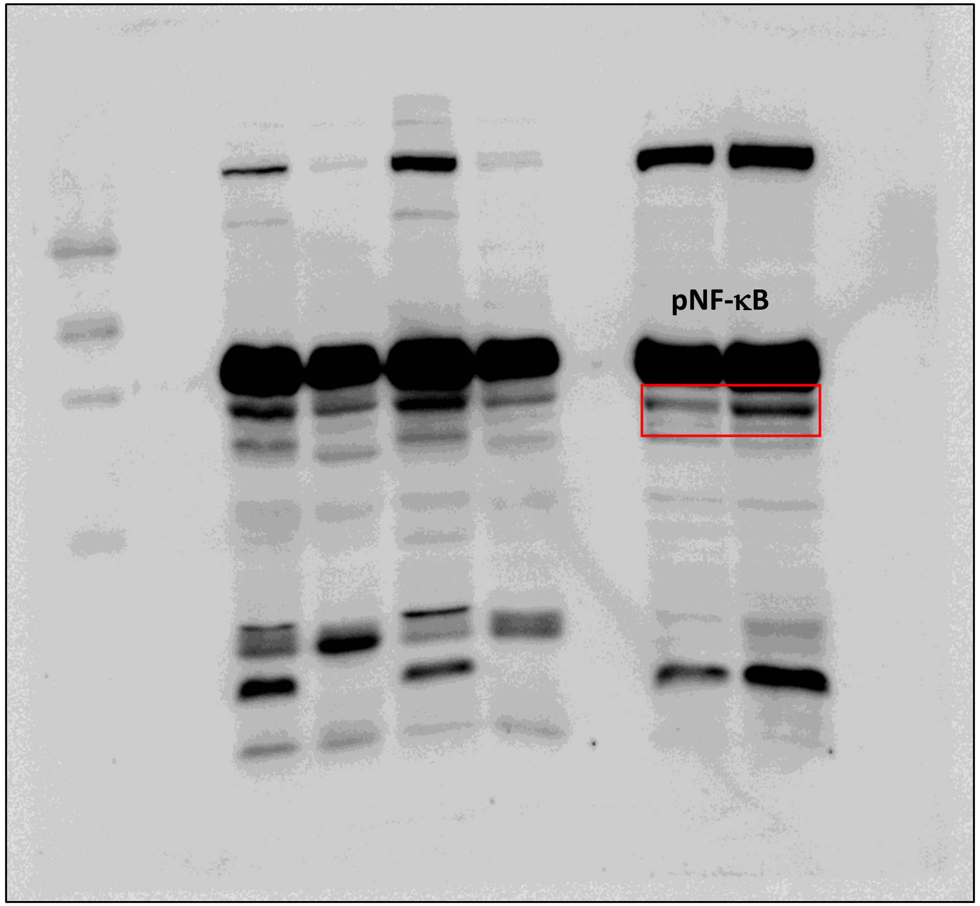

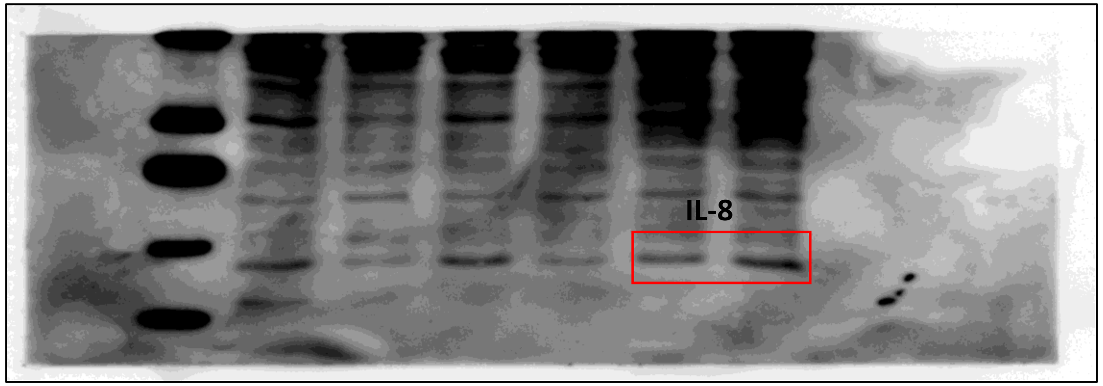

Figure 3

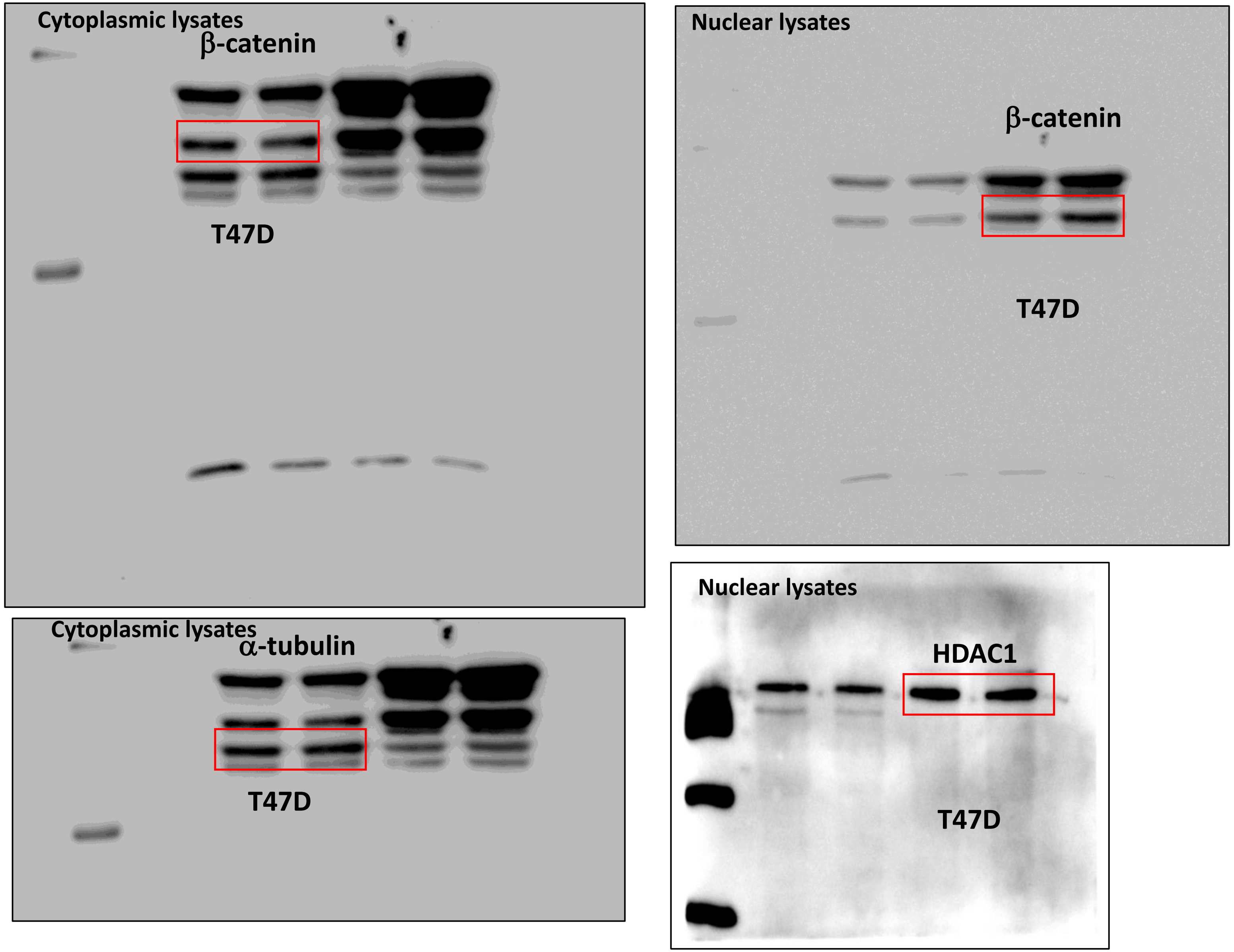
Cytoplasmic lysates
β-catenin
T47D
Nuclear lysates
β-catenin
T47D
Cytoplasmic lysates
α-tubulin
T47D
Nuclear lysates
HDAC1
T47D

Figure 3

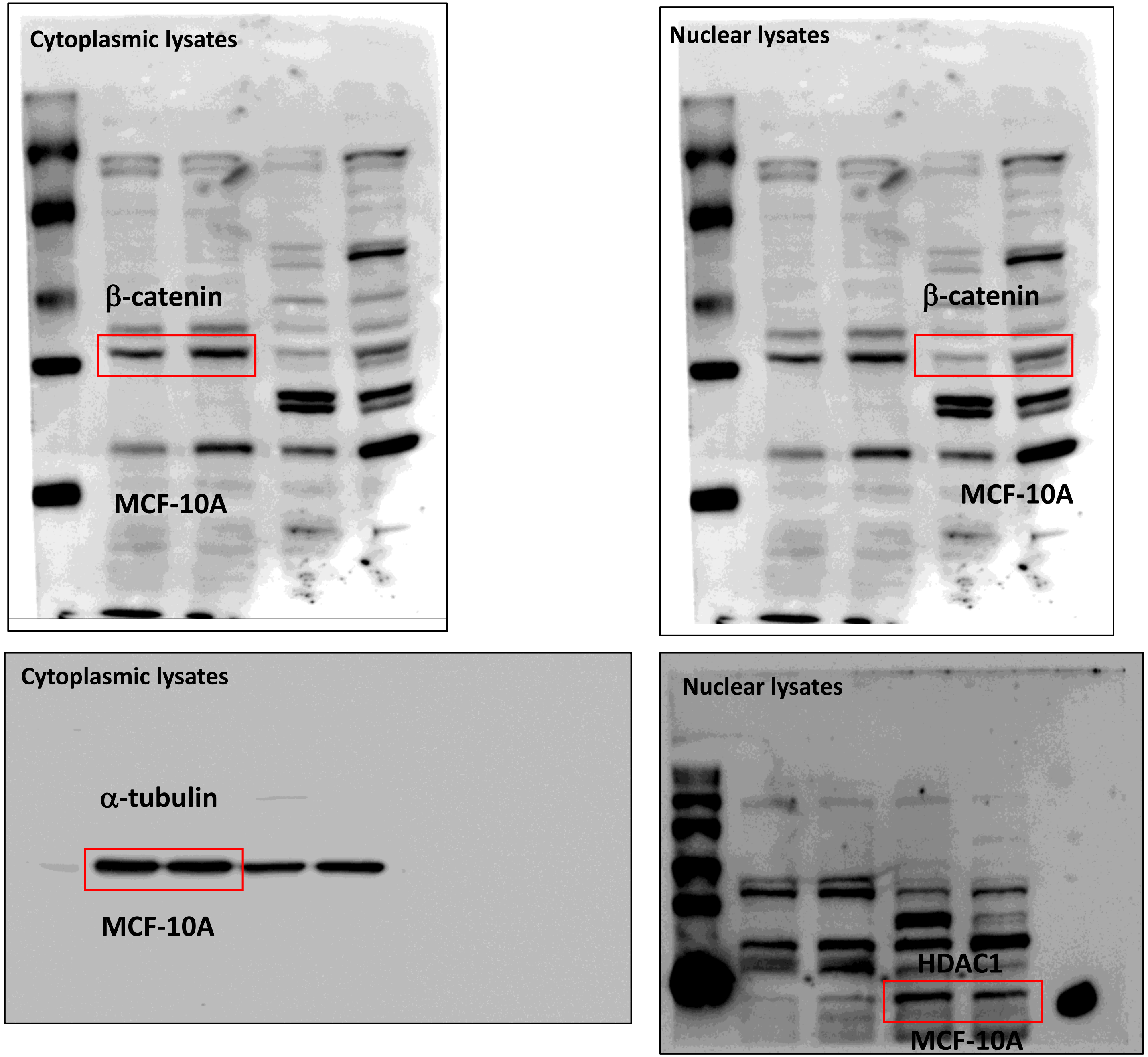
Cytoplasmic lysates
β-catenin
MCF-10A
Nuclear lysates
β-catenin
MCF-10A
Cytoplasmic lysates
α-tubulin
MCF-10A
Nuclear lysates
HDAC1
MCF-10A

Figure 7

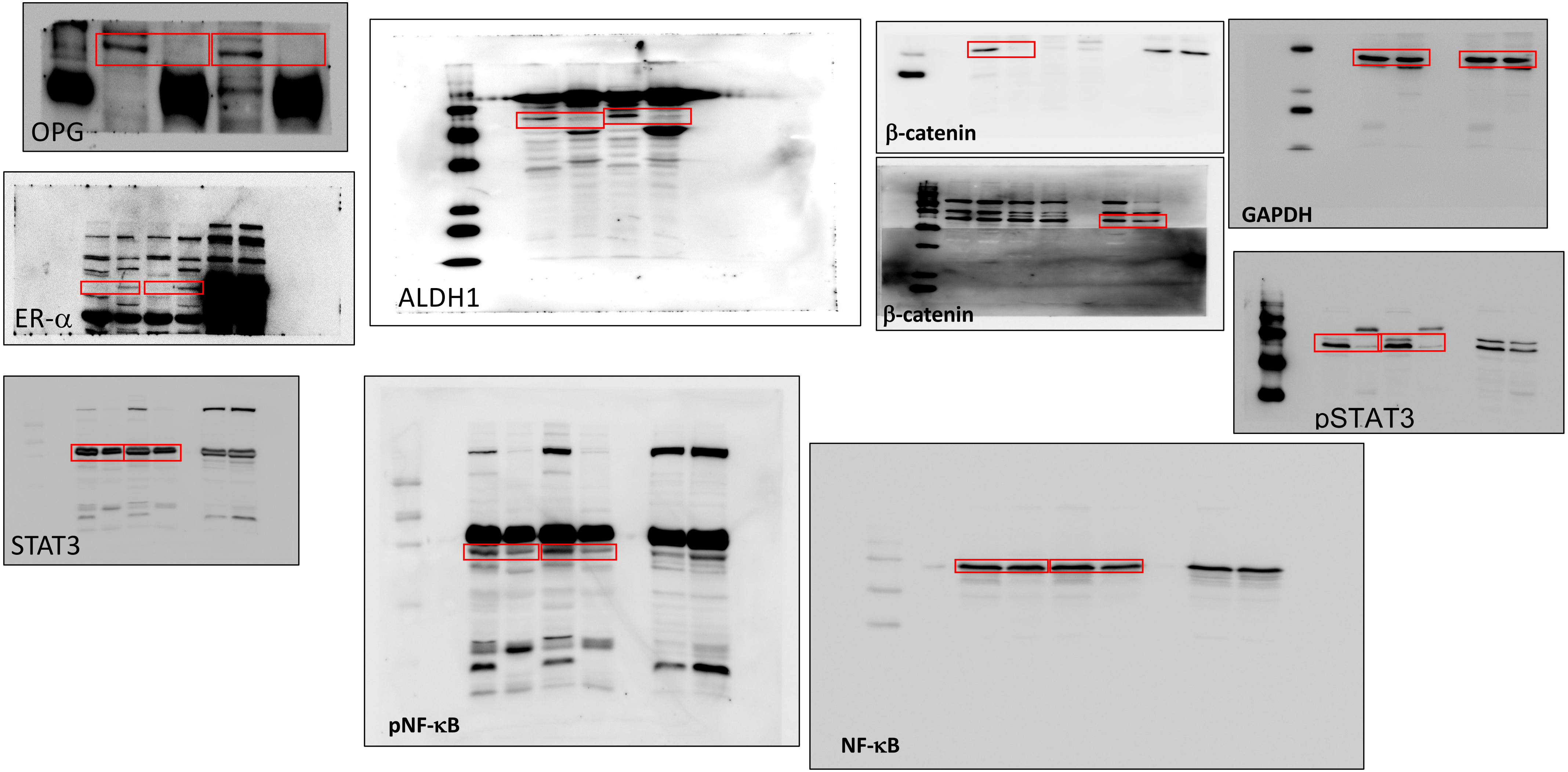
OPG
ALDH1
β-catenin
GAPDH
ER-α
β-catenin
pSTAT3
STAT3
pNF-κB
NF-κB

Figure 7

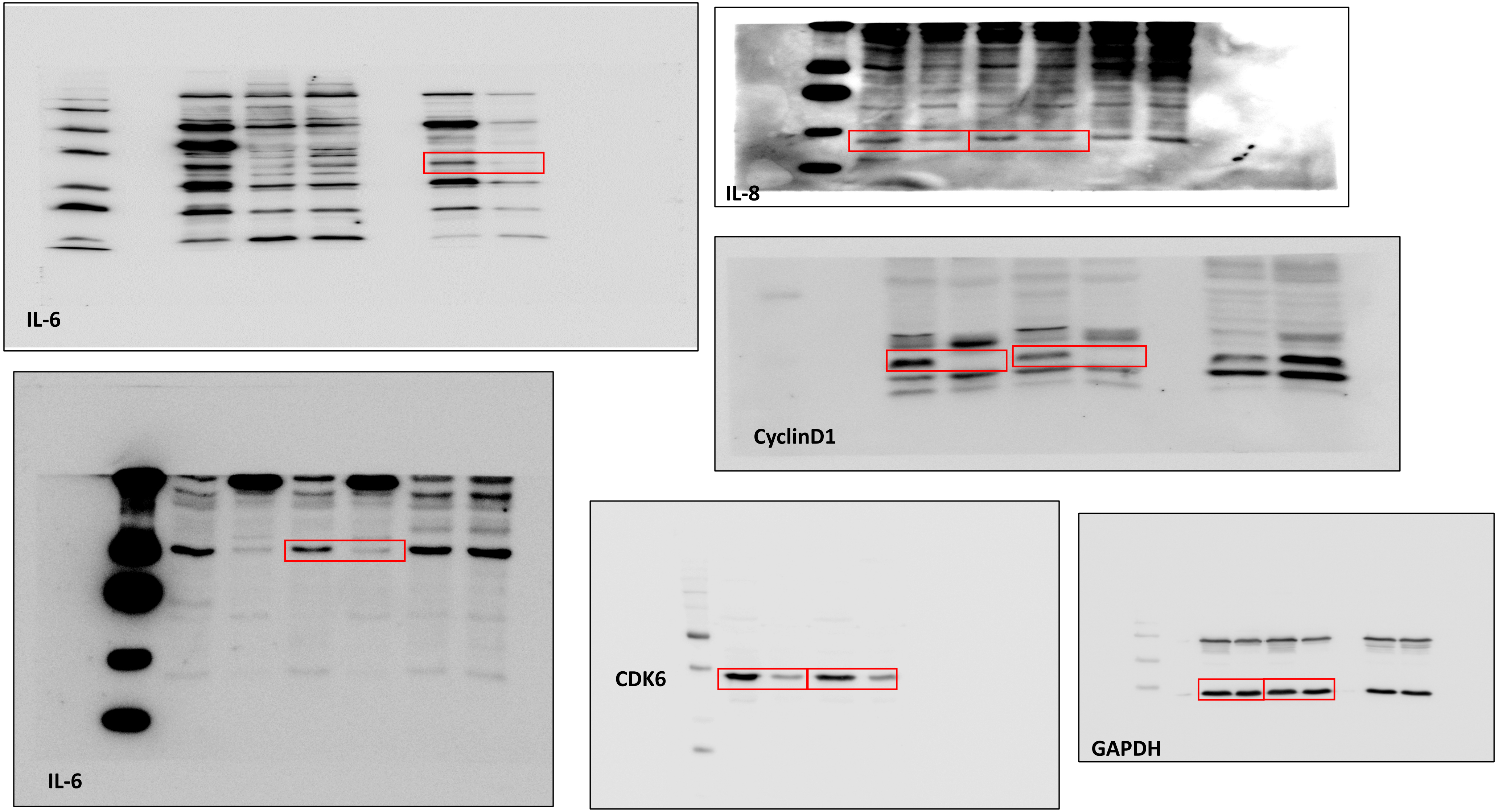

Figure 8

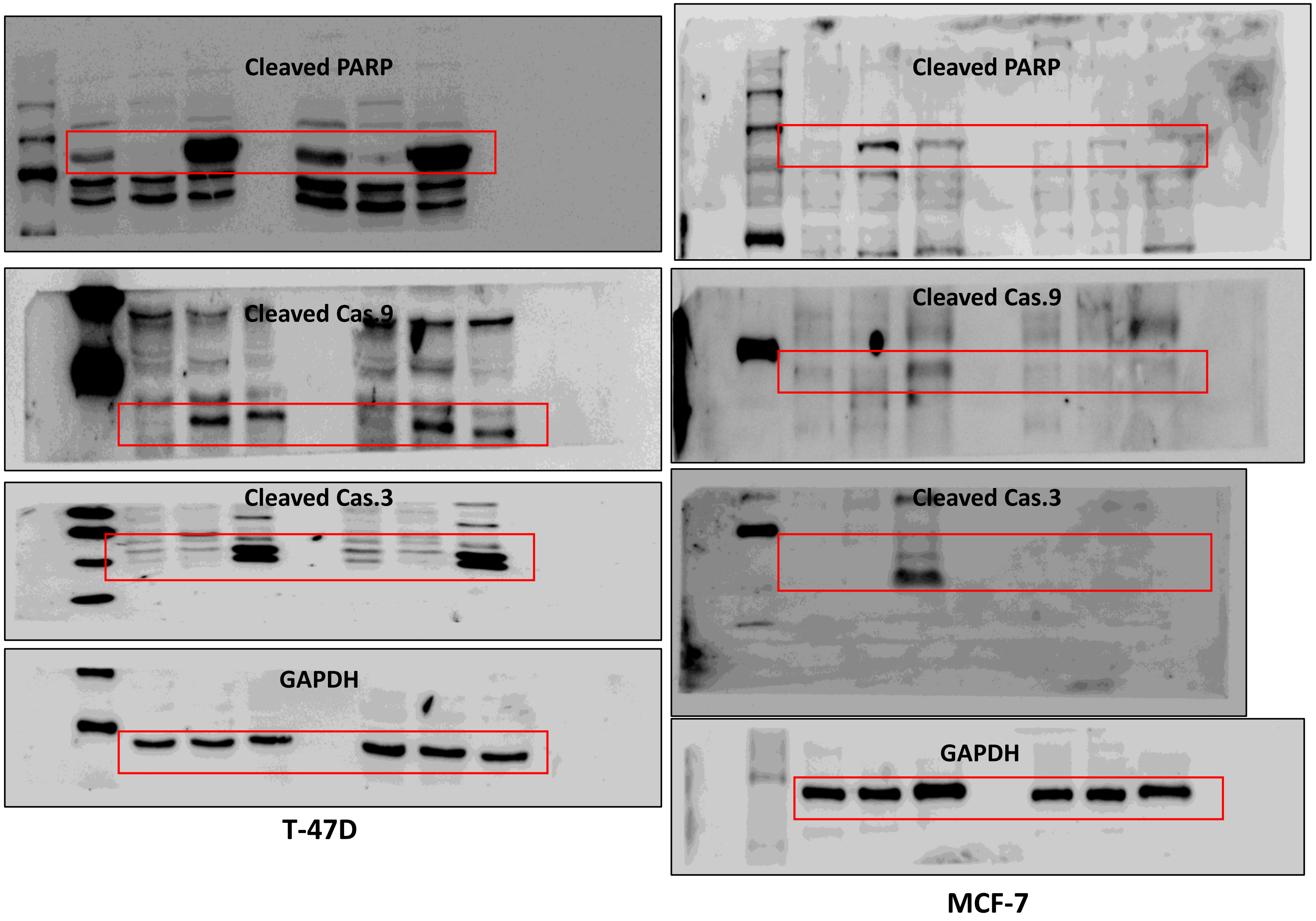
Cleaved PARP
Cleaved Cas.9
Cleaved Cas.3
GAPDH
T-47D
Cleaved PARP
Cleaved Cas.9
Cleaved Cas.3
GAPDH
MCF-7
