# Supplementary material for: Endogenous osteoprotegerin (OPG) represses ERα and promotes stemness and chemoresistance in breast cancer cells
Source: Cell Death Discov. 2024 Aug 24;10:377. doi: 10.1038/s41420-024-02151-8 (PMC11344809; doi:10.1038/s41420-024-02151-8)
Supplement: Supplementary file 2 — Supplementary information [file 41420_2024_2151_MOESM2_ESM.pdf]

SUPPLEMENTARY INFORMATION

Supplementary Table S1

Primers sequences:

Primers	Sequence	
OPG	*Forward*	*5'-AACGGCAACACAGCTCACAAG -3'*
	Reverse	*5'- TGCTCGAAGGTGAGGTTA GCA-3'*
IL-6	*Forward*	*5'-AGACAGCCACTCACCTCTTCAG -3'*
	Reverse	*5'- TTCTGCCAGTGCCTCTTTGCTG -3'*
ALDH1A1	*Forward*	*5'- TTTGGAAGATAGGGCCTGCA -3'*
	Reverse	*5'- AGGCCCATAACCAGGAACAA -3'*
CD24	*Forward*	*5'- GAGAGATAACCCTGCCCGAG-3'*
	Reverse	*5'- AAAAGAAAAGTCCGCGCCTC -3'*
CD44	*Forward*	*5'- TGGTGAACAAGGAGTCGTCA-3'*
	Reverse	*5'- GTTAAGTGTCCCAGCTCCCT-3'*
AXIN2	*Forward*	*5'-ATGCAAAAGCCACTCCAAGG -3'*
	Reverse	*5'-CTCACTCTCCAGCATCCACT -3'*
c-MYC	*Forward*	*5'-CCTGGTGCTCCATGAGGAGAC-3'*
	Reverse	*5'- CAGACTCTGACCTTTTGCCAGG-3'*
CDH1	*Forward*	*5'- CCCGCCTTATGATTCTCTGCTCGT G-3'*
	Reverse	*5'- TCCGTACATGTCAGCCAGCTTCTT G-3'*
CDH2	*Forward*	*5'- CCTCCAGAGTTTACTGCCATGAC -3'*
	Reverse	*5'- GTAGGATCTCCGCCACTGATT C -3'*
SOX2	*Forward*	*5'-GCTACAGCATGATGCAGGACCA-3'*
	Reverse	*5'-TCTGCGAGCTGGTCATGGAGTT-3'*
NANOG	*Forward*	*5'-CTCCAACATCCTGAACCTCAGC-3'*
	Reverse	*5'-CGTCACACCATTGCTATTCTTCG-3'*
ACTA2	*Forward*	*5'- CTA TGC CTC TGG ACG CAC AAC T -3'*
	Reverse	*5'- CAG ATC CAG ACG CAT GAT GGC A -3'*
CXCL12	*Forward*	*5'- CTCAACACTCCAAACTGTGCCC -3'*
	Reverse	*5'-CTCCAGGTACTCCTGAATCCAC-3'*
GAPDH	*Forward*	*5'-GAGTCCACTGGCGTCTTC-3'*
	Reverse	*5'-GGGGTGCTAAGCAGTTGGT-3'*

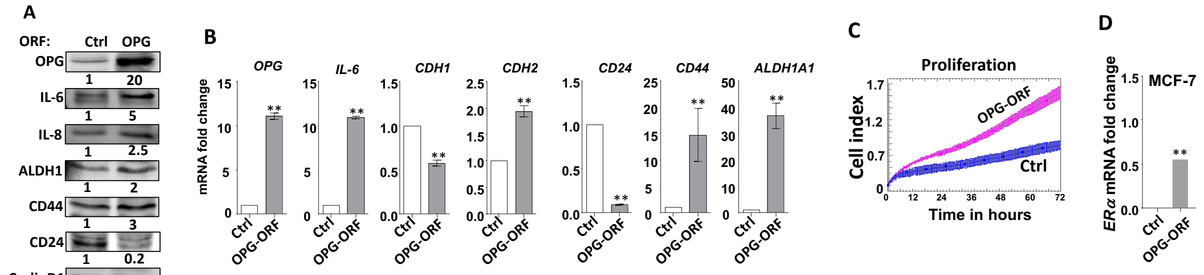

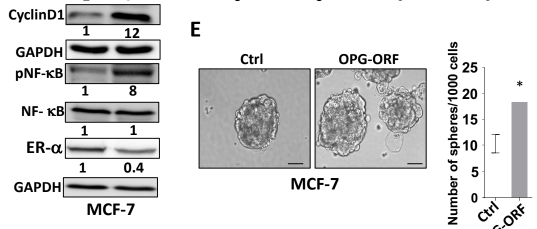

Supplementary Figure S1. OPG induces the stemness features in MCF-7 cells

(A) MCF-7 cells were transfected with a control vector (Ctrl) or a vector bearing the OPG-ORF (ORF). Whole cell lysates were prepared and were used for immunoblotting analysis using specific antibodies against the indicated proteins. The numbers below the bands indicate fold changes relative to the control (Ctrl) after correction against GAPDH. The levels of phosphorylated proteins were normalized against the total amount of their relative non-phosphorylated proteins. **(B)** Total RNA was prepared from the indicated cells, and then the expression level of the indicated genes was assessed using real-time qRT-PCR. Scale bars represent 100 µm. Error bars represent mean ± SEM (n = 3). *P ≤ 0.05; **P ≤ 0.01. (**C**) Cell proliferation abilities of MCF-7 cells bearing either a control plasmid (Ctrl) or a plasmid bearing OPG-ORF (OPG-ORF) were assessed for 72 h using the real-time xCELLigence RTCA System. Data are representative of different experiments performed in triplicate. **(D)** Total RNA was prepared, and then the expression level of the ERa gene was assessed using real-time qRT-PCR. Error bars represent mean ± SEM (n = 3). **P ≤ 0.01. **(E)** The indicated cells (1×10^3) were seeded in ultra-low attachment 96-well plate containing stem cell-specific medium (100 µl) for *in vitro* spheroid formation assay. The number of spheroids were counted with the cutoff size of 100 µm. Representative photographs of spheroids (left panel). The histogram is depicting the number of formed spheroids (right panel). Error bars represent mean ± SEM (n = 3). *P ≤ 0.05.

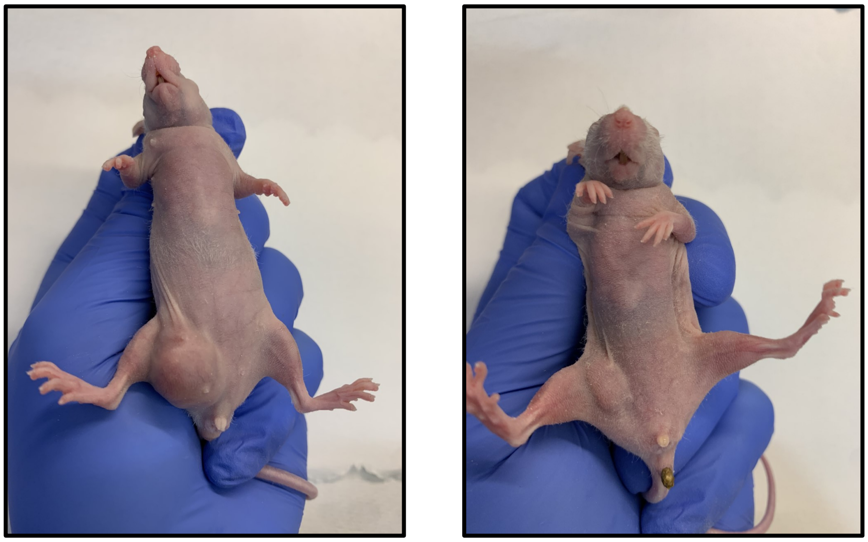

MCF-7 (5.10^6)

Supplementary Figure S2. OPG induces tumor growth *in vivo*

MCF-7 (OPG-ORF and Ctrl) cells (5×10^6) were injected under the right nipple of female nude mice (n = 5 for each inoculation).

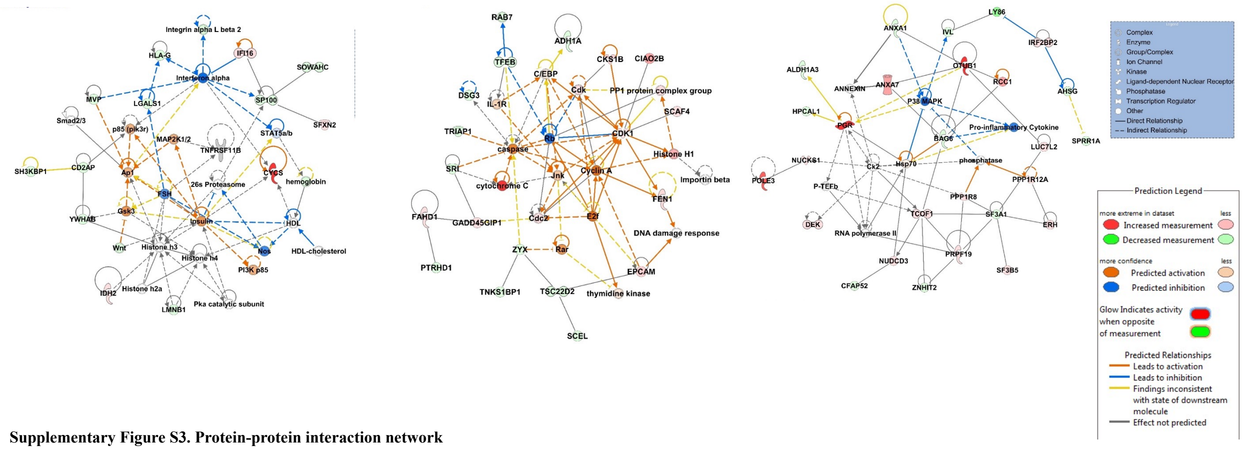

Supplementary Figure S3. Protein-protein interaction network

Protein-protein interaction network analysis of significantly dysregulated proteins (DEPs). Top scoring interaction networks with high relevancy score are shown. Straight/dashed lines represent direct/indirect protein-protein interactions, respectively. The color intensity is correlated with fold change (see legend). The significant subnetworks were identified through the use of IPA (QIAGEN Inc., https://www.qiagenbioinformatics.com/products/ingenuity-pathway-analysis).
